# Predicting chemotherapeutic drug combinations through gene network profiling

**DOI:** 10.1038/srep18658

**Published:** 2016-01-21

**Authors:** Thi Thuy Trang Nguyen, Jacqueline Kia Kee Chua, Kwi Shan Seah, Seok Hwee Koo, Jie Yin Yee, Eugene Guorong Yang, Kim Kiat Lim, Shermaine Yu Wen Pang, Audrey Yuen, Louxin Zhang, Wee Han Ang, Brian Dymock, Edmund Jon Deoon Lee, Ee Sin Chen

**Affiliations:** 1 Department of Biochemistry, National University of Singapore, Singapore; 2 National University Health System (NUHS), Singapore; 3 NUS Synthetic Biology for Clinical and Technological Innovation (SynCTI), Life Sciences Institute, National University of Singapore; 4 NUS Graduate School for Integrative Sciences and Engineering, National University of Singapore, Singapore; 5 Department of Pharmacology, Yong Loo Lin School of Medicine, National University of Singapore, Singapore; 6 Department of Pharmacy, Faculty of Science, National University of Singapore, Singapore; 7 Department of Mathematics, Faculty of Science, National University of Singapore, Singapore; 8 Department of Chemistry, Faculty of Science, National University of Singapore, Singapore; 9 School of Chemical and Life Sciences, Singapore Polytechnic, Singapore; 10 Changi General Hospital, Ministry of Health, Singapore

## Abstract

Contemporary chemotherapeutic treatments incorporate the use of several agents in combination. However, selecting the most appropriate drugs for such therapy is not necessarily an easy or straightforward task. Here, we describe a targeted approach that can facilitate the reliable selection of chemotherapeutic drug combinations through the interrogation of drug-resistance gene networks. Our method employed single-cell eukaryote fission yeast (*Schizosaccharomyces pombe*) as a model of proliferating cells to delineate a drug resistance gene network using a synthetic lethality workflow. Using the results of a previous unbiased screen, we assessed the genetic overlap of doxorubicin with six other drugs harboring varied mechanisms of action. Using this fission yeast model, drug-specific ontological sub-classifications were identified through the computation of relative hypersensitivities. We found that human gastric adenocarcinoma cells can be sensitized to doxorubicin by concomitant treatment with cisplatin, an intra-DNA strand crosslinking agent, and suberoylanilide hydroxamic acid, a histone deacetylase inhibitor. Our findings point to the utility of fission yeast as a model and the differential targeting of a conserved gene interaction network when screening for successful chemotherapeutic drug combinations for human cells.

Cancer is a global health problem and there is a critical need for new advancements in its management and treatment if we are to meet the projected increase in new cases^1^. One important challenge facing clinicians is how to determine the most appropriate treatment strategy for patients. Thus far, chemotherapy has remained the mainstay for cancer management^2^. Yet, most of the widely employed anticancer drugs used in the clinic are cytotoxic, harbor a relatively narrow therapeutic window, and/or pose a risk for the development of severe side effects that can potentially jeopardize treatment outcomes^3^. Despite the toxicity experienced by patients, evidence shows the importance of maintaining a consistent dosage of chemotherapeutic agents within the body over the therapeutic period to kill cancer cells. This high, constant drug insult often induces selection pressure, which encourages the proliferation of certain cells that exhibit increased counter-chemotherapeutic responses, thus leading to chemoresistance^4^.

Drug responsiveness, cytotoxic side effects, and the onset of resistance are linked to the ‘genetic makeup’ of the patient but this concept itself also remains poorly defined^5^. Recent breakthroughs in genome-wide profiling have uncovered an intimate correlation between molecular signatures, such as gene expression, and patient drug responsiveness, and these findings provide support for the hypothesis that molecular processes are drivers of carcinogenesis that may be employed as discriminating criteria to further fine-tune treatment options^2^.

Numerous genes bearing significance in carcinogenesis or contributing significantly to various cancer signatures have been described as hallmarks of cancers^6^ and have been deemed as obvious ‘druggable’ targets for chemotherapy^7^. Genome-based profiling has also revealed a large collection of genes, many of which have, as yet, undefined connections to carcinogenesis. These genes do not function alone but, rather, act in synergy with other so-called partner genes to control heritable attributes^8^. Some of these interactions have been characterized through direct protein–protein binding studies but most have been defined through genetic means^9^. Although the functions of individual genes may not be resolved, it is clear that these synergistic relationships among genes form a ‘safety net’ to ensure cell survival, especially following environmental insults by agents such as cytotoxic chemotherapeutic compounds^10^. In recent years, considerable attention has been given to these genetic interactions, as they underpin the synthetic lethality approach, which is considered to be able to predict the precise combination of drugs that will improve the efficacy of chemotherapeutics^11^. Despite the potential utility of this approach, elucidating genetic connectivity in human cells remains a technically and financially challenging task^12^.

Clinical chemotherapeutic applications involve drug combinations of typically three to four drugs^13^,^14^, and determining the most suitable drug combination profoundly affects chemotherapeutic success. Drugs that possess varied modes of action would be expected to counteract different mechanisms of cellular resistance, and, by that reasoning, should provide efficient or even better therapeutic outcomes when combined rather than applied singly. To this end, we herein describe an approach to predict the efficacy of chemotherapeutic drug combinations that will act to kill human cancer cells in a cooperative manner. This approach is based on an overlapping drug resistance network elucidated using fission yeast (*Schizosaccharomyces pombe*) as a surrogate model. Drugs with a smaller overlap in the resistance gene network are considered to have different mechanisms of action and thus, in view of our reasoning above, would be expected to demonstrate a stronger cooperative effect with the target drug (doxorubicin in our case).

To provide evidence for the utility of such a work flow, we interrogated our previously identified doxorubicin resistance (DXR) genetic network and the intersection of doxorubicin with six other drugs: hydroxyurea (HU), an inhibitor of the deoxyribonucleotide biosynthesis enzyme ribonucleotide reductase (RNR)^15^; methyl methanesulfonate (MMS), an alkylating agent that causes nucleotide base damage and impedes the progress of the DNA replication fork^16^; camptothecin (CPT), a topoisomerase I inhibitor^17^; thiabendazole (TBZ), a microtubule destabilizing agent^18^; cisplatin, an intra-DNA strand cross-linker and blocker of transcription^19^,^20^ and suberoylanilide hydroxamic acid (SAHA), a histone deacetylase inhibitor^21^. With the exception of TBZ, which targets mitotic spindle microtubules, the remaining drugs are reported to induce DNA aberrations that cause DNA damage^15^,^16^,^17^,^18^,^19^,^20^,^21^,^22^,^23^.

Profiling performed in fission yeast, however, showed that the genes and networks that function to respond to HU, MMS and CPT—initially chosen based on differential mode of action—showed an unexpectedly high degree of overlap. SAHA and cisplatin, on the other hand, exhibited unique gene profiles. These drugs were further shown to act cooperatively with doxorubicin to result in preferential growth retardation in several DXR mutants and interestingly, also in human cancer cells. Thus, the degree of similarity in synthetic lethal drug hypersensitivity profiles and network overlap in fission yeast is useful to narrow down the potential drug candidates and select the best combination(s) that could be subsequently applied to a human cancer cell model.

## Results

### Study Design Overview

We recently performed a screen for genes that confer doxorubicin resistance (DXR) using fission yeast (*Schizosaccharomyces pombe*) as a model, and found that a network of genes protected cells against drug cytotoxicity^24^,^25^. We noted that many cells with mutations in these DXR genes exhibited hypersensitivity to a range of drugs, not specifically doxorubicin^23^,^26^,^27^, and this pointed to a potential overlap in drug resistance mechanisms and/or the existence of a genetic network counteracting multiple cytotoxic agents in fission yeast.

To assess this potential link between the DXR cellular network and that of other drugs, we tested the hypersensitivity of 90 DXR mutant strains with each of the following six drugs: HU, MMS, CPT, TBZ, cisplatin and SAHA. We chose again to use fission yeast cells to test the drug combinations in these 90 genes because of the time and cost efficiencies afforded by this model over human cells.

We serially diluted exponentially growing DXR mutant cells and manually spotted the cultures onto plates incorporated with various concentrations of each of the six drugs: 0, 2, and 4 mM of HU; 0, 6, 8, 10, and 12 μM of CPT; 0%, 0.001%, 0.002%, 0.005%, 0.01%, and 0.02% of MMS; 0, 8, and 10 μg/ml of TBZ; 0, 1.2, and 1.6 mM of cisplatin; 0 and 10 mM of SAHA. The following null mutants were employed as positive controls for our drug-hypersensitivity assays: for HU, MMS and CPT, we used the DNA damage checkpoint kinase *rad3* (Δ*rad3*) (Supplementary Fig. 1–3); for TBZ and cisplatin, α-tubulin *nda3* (*nda3-KM311*) (Supplementary Fig. 4, 5); and for SAHA, a type I histone deacetylase complex *alp13* (Δ*alp13*) (Supplementary Fig. 6)^18^,^28^,^29^,^30^. In all cases, an isogenic, prototrophic wild-type (WT) strain was used as a negative control and also as an indicator that the drug concentrations used were within the physiological range and did not affect WT cell growth^24^.

### Classification of drug hypersensitivity mutants using sensitivity scores

**Figure 1:**
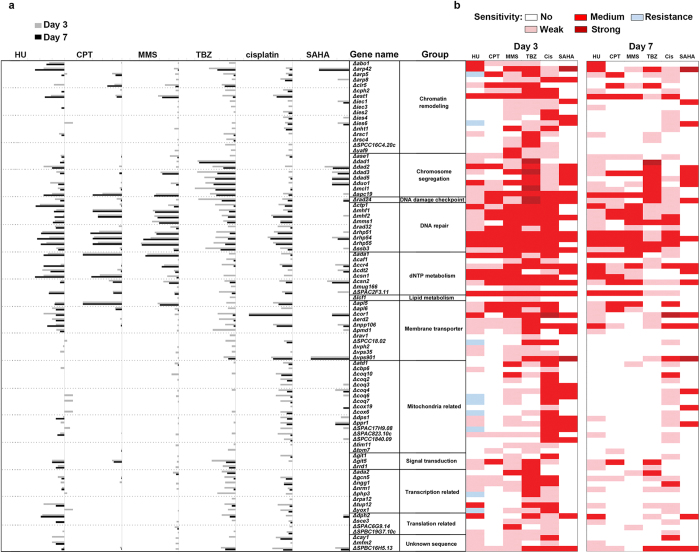
Sensitivity score (s-score) of the doxorubicin resistance (DXR) mutants obtained at different durations of drug exposure. (**a**) Grey and black bars indicate days 3 and 7, respectively, after drug exposure. (**b**) Level of hypersensitivity. Dark red, high; red, medium; pink, low; white, not sensitive; and light blue, resistant. DXR genes that were disrupted in the null mutants are listed. HU: hydroxyurea, CPT: camptothecin, MMS: Methyl methanesulfonate, TBZ: thiabendazole, Cis: cisplatin, SAHA: suberoylanilide hydroxamic acid.

The growth of the various strains was documented during the intermediate and stationary phases (days 3 and 7 after drug treatment, respectively (Supplementary Fig. 1–6)). Growth was quantified using a sensitivity score (s-score), which accounted for the relative fold sensitivity of a specific DXR mutant over the range of the drug concentrations tested as compared with that of untreated cells. Relative changes in fold sensitivity were normalized by comparing the WT strain on plates with drugs over those without. A negative s-score depicts drug sensitivity, whereas a positive s-score denotes drug resistance. An s-score of 0 was obtained when the growth of a particular strain resembled that of WT cells (Supplementary Fig. 7). Cells that showed less than 10-fold sensitivity at only one drug concentration were classified as weakly sensitive mutants (pink, Fig. 1b), whereas cells exhibiting 10-fold or greater sensitivity for all drug concentrations were classified as strongly sensitive (dark red, Fig. 1b); the values in between (from > 10-fold in one concentration up to 10-fold at all concentrations) were deemed to have a medium sensitivity (red, Fig. 1b).

### Multidrug resistance is controlled by regulators of various DNA-related processes

We noted that almost all DXR mutants showed hypersensitivity to the drugs tested, with very few drug-resistant phenotypes (Fig. 1a). In strains showing drug resistance, this resistance was only transiently observed during the intermediate growth stage (day 3) when the strains grew faster relative to that of WT, reminiscent of a resistance-like growth pattern (Fig. 1a). However, upon further incubation, these ‘transiently resistant’ strains (Δ*arp5*, Δ*ies6*, Δ*SPCC18.02*, Δ*coq6*, Δ*coq7*, Δ*cox6,* Δ*SPAC17H9.08*, Δ*php3*, and Δ*yox1*; Fig. 1) were shown to be hypersensitive, but only to HU.

A previous study used genome-wide synthetic lethality screens to test HU, CPT, and MMS in fission yeast^26^. Consistent with this previous report, we observed that Δ*rhp51*, Δ*rhp54*, Δ*rhp55*, Δ*rad32*, Δ*mhf1*, Δ*rad24*, and Δ*cdt2* showed sensitivity towards HU, MMS and CPT (Fig. 1a)^26^. Interestingly, these mutants also exhibited sensitivity towards doxorubicin, TBZ and SAHA, indicating that these genes may be involved in a fundamental mechanism to regulate multidrug resistance (Fig. 1b). Ontologically, these seven genes function in homologous recombination (HR) repair, checkpoint signaling and also nucleotide synthesis^31^,^32^, suggesting that these mechanisms coordinate resistance against agents that induce chromosomal aberrations (Fig. 1b). The sensitivity towards HU, MMS, and CPT is consistent with the roles of these genes in the management of DNA damage. These mutants also exhibited strong hypersensitivity towards TBZ, a microtubule destabilizing agent that, conceptually, does not affect DNA integrity directly^18^. Although SAHA is a histone deacetylase inhibitor (HDACi), it has also been reported to induce DNA double-stranded breaks (DSBs) in humans^22^,^33^, and this is probably due to the role of HDAC in chromatin compaction to safeguard cells against unwarranted access by DNA damaging agents^29^.

A loss of *est1*—a telomerase regulator that maintains the length of telomeric DNA at the chromosomal ends—showed sensitivity to most of the drugs tested, again, with the exception of SAHA (Fig. 1b). This hypersensitivity remained equally high on days 3 and 7, suggesting that the mutants may lose viability upon exposure to the drugs. This phenotype is highly reminiscent of that of DNA damage checkpoint mutants; for example Δ*rad3* on HU plates^28^,^30^ (Supplementary Fig. 1). Overall, these findings are consistent with the previous hypothesis^34^ that genomic instability at the telomere underlies cytotoxicity of chemotherapeutic drugs towards human cancer cells.

### Overlap between gene sets regulating resistance toward multiple drugs

We next classified DXR genes by the number of drugs they showed sensitivity to at both the intermediate (day 3) and stationary (day 7) growth phases. Interestingly, in the intermediate growth phase, all mutants—aside from those that showed a resistance-like growth to HU (Fig. 1a)—showed sensitivity toward at least one drug (day 3, Fig. 1b), suggesting that DXR genes are part of a centralized network that confers resistance to cytotoxic agents. Furthermore, this differential sensitivity pointed toward the existence of sub-divisions within the centralized network.

We surmised that the genetic overlap seen for this subset of drugs may point to a similar mode of action for the drugs, despite choosing drugs with apparently disparate actions. We noted that at day 7, 13 genes showed no sensitivity toward all of the additional drugs tested aside from doxorubicin, whereas 26 genes remained hypersensitive to only one drug in addition to doxorubicin; this cohort comprised 43.3% (39/90) of the total number of DXR genes examined. It is possible that these genes are involved in a sub-network that responds to doxorubicin. Interestingly, many of the genes in this sub-cluster encode enzymes of the coenzyme Q10 synthesis pathway (*coq2*^+^, *coq4*^+^, *coq6*^+^, *coq7*^+^, *dps1*^+^)^35^, suggesting that coenzyme Q10 may play an specific role in conferring resistance to doxorubicin.

### Doxorubicin resistance gene network

**Figure 2:**
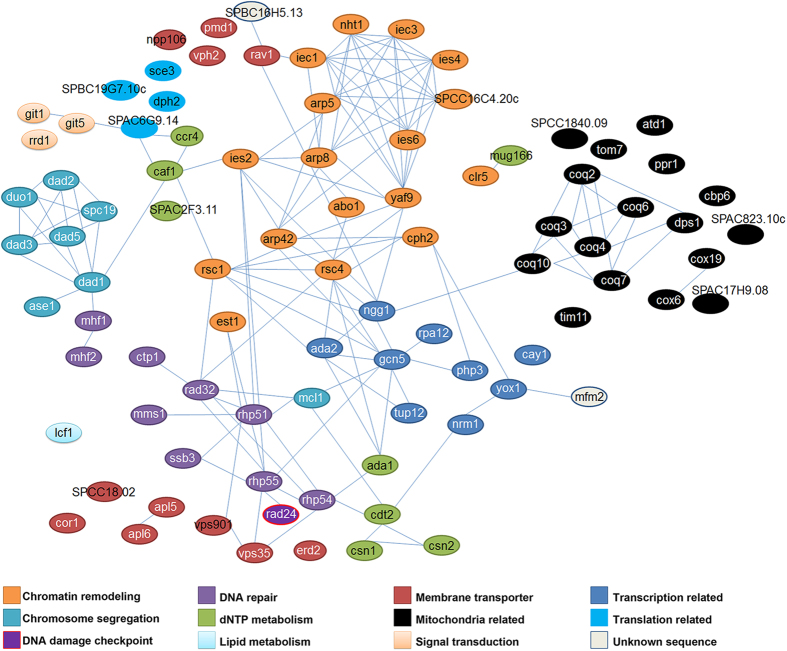
Gene network of doxorubicin resistance (DXR) genes. Linkages between the DXR genes were obtained using String ver. 9.1. The genes are color-coded according to their ontological/functional classification.

We next investigated connectivity among the DXR genes by assessing published physical and genetic interactions using the online bioinformatics tool, String (version 9.1)^36^. With the exception of *lcf1*^+^, *apl5*^+^, *apl6*^+^, *erd2*^+^ *sce3*^+^, *dph2*^+^, *npp106*^+^, *vph2*^+^, *pmd1*^+^, *mug166*^+^, *cor1*^+^, *clr5*^+^, *ppr1*^+^, *cay1*^+^, *atd1*^+^ and several uncharacterized coding sequences (*SPCC18.02*, *SPBC19G7.10c*, *SPAC17H9.08* and *SPAC823.10c*), most of the genes isolated in our DXR screen can be assimilated into an extensive network that conceptually depicts, at least in part, a centralized multidrug resistance (MDR) network (Fig. 2). These connections are functionally defined and mostly depict physical interactions as curated by String^36^. However, they also comprise genetic interactions and catalysis mechanisms involved in similar biochemical pathways, particularly for ubiquinone^35^.

The DXR gene network consists of several sub-clusters that can be grouped according to ontological pathways: mitochondrial function/coenzyme Q10 biosynthesis; chromatin remodeling, particularly highlighting Ino80 complex components; chromosomal segregation, primarily the DASH complex; membrane-associated transport; nucleotide metabolism; signal transducers (a small cluster); DNA damage response; transcriptional regulation; and protein translation (Fig. 2). We noticed that the DXR network was centrally connected by several ‘hubs’ encompassing the histone acetyltransferase SAGA complex and chromatin remodeling factors (but excluding the Ino80 complex) (Fig. 2). S-scores of Δ*gcn5* at day 3 indicated that it exhibited high sensitivity towards cisplatin and TBZ but was weakly affected by HU and CPT, and not sensitive to MMS and SAHA (Fig. 1b). By day 7, Δ*gcn5* cells exhibited a weak sensitivity to the drugs (Fig. 1b). These findings suggest that Δ*gcn5* serves a supportive role that involves decondensing the chromatin to facilitate transcribed RNA-templated HR repair^37^ or to regulate DNA damage checkpoint activation^38^.

HR DNA repair factors also assumed a focal position, especially Rhp51 (Fig. 2). Unlike Δ*gcn5*, Δ*rhp51* showed strong hypersensitivity towards all six drugs tested on both days 3 and 7 (Fig. 1b, 2b). Rhp51-like protein is required to repair DSBs and enforce DNA damage tolerance during S-phase of the cell cycle^39^; this likely explains the importance of HR proteins in facilitating resistance to most drugs used in this study. Mutants with compromised HR integrity also showed hypersensitivity to TBZ, which disrupts microtubule assembly; this hypersensitivity is probably based on the connection between the DNA replication checkpoint and the spindle assembly checkpoint^40^.

### Sub-clusters of the doxorubicin gene network are also required to respond to other cytotoxic agents

**Figure 3:**
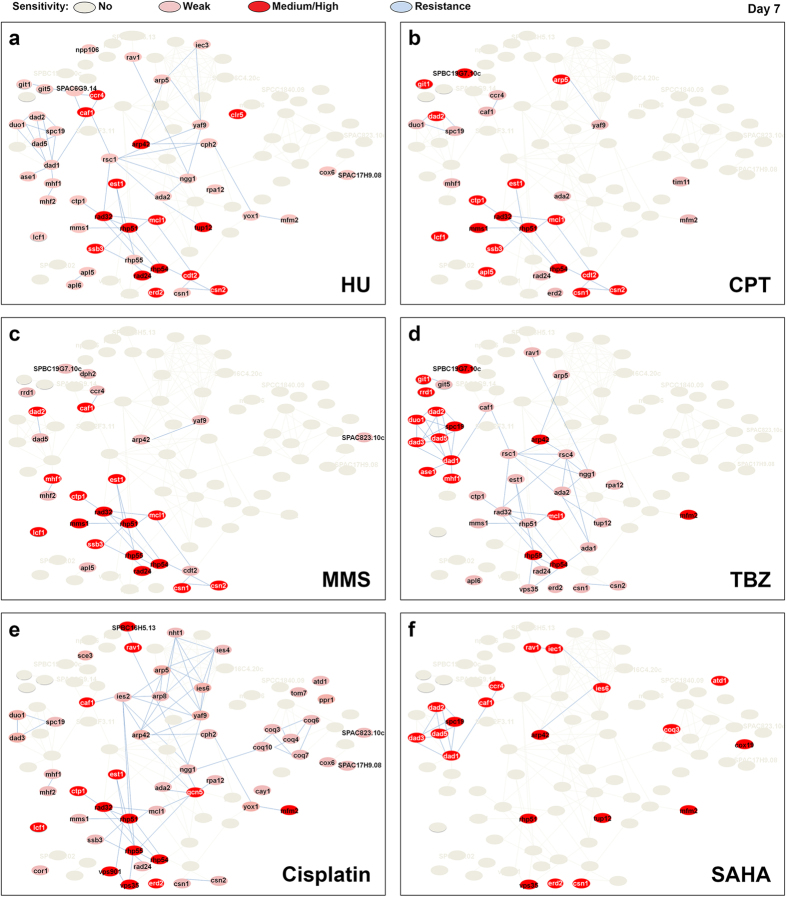
Overlap in drug resistance network between the tested drugs and doxorubicin. The DXR mutants that remained hypersensitivity on day 7 upon exposure to (**a**) hydroxyurea (HU), (**b**) camptothecin (CPT), (**c**) methyl methanesulfonate (MMS), (**d**) thiabendazole (TBZ), (**e**) cisplatin, and (**f**) suberoylanilide hydroxamic acid (SAHA). Strains that showed sensitivity across all drug concentrations tested or only on one of the tested concentrations: red, high to medium sensitivity; pink, weak sensitivity.

Next, we sought to identify mutants that showed concomitant hypersensitivity to doxorubicin and one or more of the other tested drugs. This was achieved by determining any overlap in the drug-resistance network. Null mutants that were hypersensitive (red: medium-strong; pink: weak sensitivity, Fig. 3a–f) to HU (Fig. 3a), CPT (Fig. 3b), MMS (Fig. 3c), TBZ (Fig. 3d), cisplatin (Fig. 3e), or SAHA (Fig. 3f) were overlaid with the DXR gene network to highlight a genetic overlap. We found a significant overlap of DXR nodes for HU, CPT and MMS—each of which disrupt DNA replication (p<0.0001)^15^,^16^,^17^,^41^ (Fig. 3a-c). This overlap lends further support to the theory that DXR genes belong to a centralized MDR network^42^. We noted that cisplatin also shared considerable overlap with the HU/CPT/MMS cluster; albeit, with more variation (Fig. 3f).

### Cooperative effect among doxorubicin, SAHA and cisplatin in fission yeast

Given the significant gene overlap noted among several of the drugs, we further interrogated the MDR drug response network to predict an efficient drug combination that may be used to sensitize human cancer cells toward doxorubicin. We first spotted serially diluted WT fission yeast cells on media incorporated with increasing concentrations of doxorubicin and sub-lethal doses of MMS (0.01%), TBZ (8 μg/ml), HU (2 mM), CPT (8 μM), cisplatin (1.2 mM), or SAHA (5 mM) as a control (Supplementary Fig. 8). Overall, WT cells were not affected by the drug treatments. We observed a slight growth reduction with doxorubicin, CPT and SAHA on day 3 (Supplementary Fig. 8a, day 3) but cells were generally unaffected, as growth on day 7 was similar between treated and untreated cells (Supplementary Fig. 8b, day 7). No change was observed for cells treated with HU, CPT, MMS or TBZ.

We further assessed the synergism noted for the combination of doxorubicin, SAHA and cisplatin by repeating the serial dilution and spotting assays using several DXR null mutants: Δ*rav1*, Δ*rhp51*, Δ*vps35*, Δ*caf1* and Δ*tim11*. These genes encode for a vacuolar ATPase assembly factor (Δ*rav1*), a HR factor (Δ*rhp51*), a subunit of endosome sorting (Δ*vps35*), a protein of the CCR/NOT deadenylase complex (Δ*caf1*), and a mitochondrial ATPase complex protein (Δ*tim11*)^32^,^43^,^44^,^45^. In human cancers, these proteins have been linked with drug resistance and/or cancer cell proliferation^25^,^46^,^47^,^48^,^49^. We found that Δ*rhp51* cells showed exceedingly high sensitivity to doxorubicin as well as to that of other drugs, and therefore any synergistic effect was likely masked. The Δ*rav1* null mutant, in comparison, showed particular hypersensitivity to cisplatin, again preventing the identification of any cumulative drug effects. Interestingly, the remaining three mutants (Δ*vps35*, Δ*caf1* and Δ*tim11*) showed prominent attenuation of a growth defect when the cells were treated with the triple combination of doxorubicin, cisplatin and SAHA, thus demonstrating a synergistic effect for the drugs and a role for these genes (Supplementary Fig. 9).

### Sensitization of human cancer cells to doxorubicin by SAHA and cisplatin

Next we tested whether the cooperative effect of doxorubicin, cisplatin and SAHA could be recapitulated in human cancer cell models and thus confirm whether fission yeast serves as a suitable model for the screening of chemotherapeutic drug combinations. Others have reported in the past that mutations in genes of the vacuolar sorting pathway are connected to gastric cancer occurrence^46^, so we tested our drug combination first on gastric adenocarcinoma (AGS) cells. Indeed, gastric cancers are treated clinically with a chemotherapeutic regime containing doxorubicin and cisplatin^50^.

**Figure 4:**
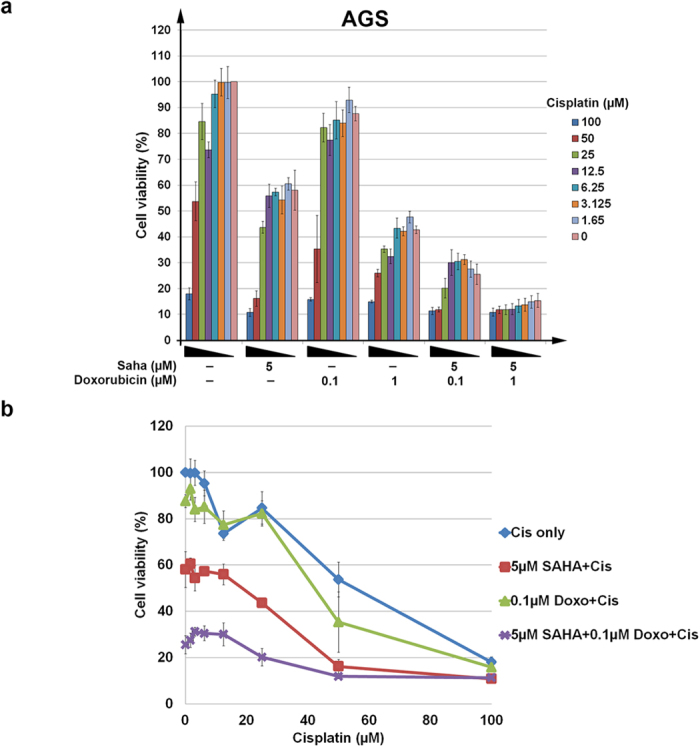
Sensitization of human gastric adenocarcinoma (AGS) cells to doxorubicin via concurrent treatment with cisplatin and SAHA. (**a**) Cells were co-treated with varying concentrations of cisplatin in the presence of 5 μM SAHA, or 0.1 or 1 μM doxorubicin or with a triple combination of cisplatin, 5 μM SAHA and 0.1 or 1 μM doxorubicin. (**b**) Dose response effect on the viability of AGS cells was analyzed. Cells were treated with varying concentrations of cisplatin alone (blue), in combination with 5 μM SAHA (red) or 0.1 μM doxorubicin (green), or both 0.1 μM doxorubicin and 5 μM SAHA (purple).

AGS cells were treated in single, double or triple combinations of cisplatin (0 μM to 100 μM), SAHA (5 μM), and doxorubicin (0.1 or 1 μM) (Fig. 4). Cisplatin alone induced a concentration-dependent cytotoxicity in AGS cells and showed an additive effect with doxorubicin, particularly at higher concentrations of doxorubicin (1 μM); lower doxorubicin concentrations (0.1 μM) had little to no effect (Fig. 4, beige bars). SAHA treatment alone reduced AGS cell viability, and this was further decreased with the addition of doxorubicin. The triple combination killed the cells and affected the entire dose-response curve (Fig. 4b). This synergism among the three drugs was particularly apparent at 25–50 μM cisplatin, 0.1 μM doxorubicin and 5 μM SAHA (Fig. 4b). Thus, as with fission yeast cells, the three drugs worked cooperatively to kill gastric adenocarcinoma cells (Fig. 4). We also observed a similar cooperative effect in human cervical carcinoma (HeLa) cells (Supplementary Fig. 10a,b) but not in the non-cancerous embryonic kidney HEK293 cells (Supplementary Fig. 11a,b). This observation is reminiscent of the lack of effect of the three-drug combination on WT fission yeast cells (Supplementary Fig. 8). Taken together, our results show that fission yeast can be used as a surrogate model to derive effective chemotherapeutic combinations to target human cancer cells.

## Discussion

In this report, we show that large-scale genetic synthetic lethality screening in fission yeast can be used to increase the reliability of predicting the synergistic effect of multiple drugs when targeting human cancer cells. This study provides a proof-of-concept that three drugs—doxorubicin, cisplatin and SAHA, which show a low degree of overlap among gene networks in fission yeast cells—can synergistically work to affect cell growth.

With the completion of sequencing for many organisms, especially that of yeast, the ability to interrogate the functional implications of individual genes to a phenotype has revolutionized genome-based studies^25^,^51^. Large-scale genetic interaction assays have made it possible to model the lives of eukaryotic organisms in order to identify the complicated connectivity among genes and study how this interactivity constitutes a network that determines the phenotype^52^,^53^; in this case, chemotherapeutic resistance. The study of genetic interactions has gained momentum, and the revelation of the ‘interactome’^54^ has shed light on how multiple components can be regulated concurrently. It has also (fortunately) revealed that there are rules that govern how the structure and topology of genetic networks operate, and studies show that genes within the network often form a relatively restricted set of positive and negative interactions^12^. Cross-species studies have also identified conservation of a fundamental network that is universal in higher eukaryotes, including humans, and it has been determined that this network shares, unexpectedly, great overlap with that of single cellular eukaryotes, such as yeast^25^,^55^. Given that toxin responses are fundamental to all cells, it is likely that this forms the basis for the conserved mechanism of drug resistance observed between fission yeast and human cells. Thus, it is plausible to take advantage of the commonality amongst eukaryotic cells to understand the molecular mechanism of resistance to chemotherapeutic agents.

The results presented here support our expectation that large-scale synthetic lethality network elucidation in unicellular eukaryotes (such as fission yeast) would be a suitable model with which to predict a pharmacological regimen for sensitizing human cancer cells to chemotherapeutic agents. Gene knockout screening in human cell models is typically costly and time consuming. By comparison, our synthetic lethality workflow with fission yeast demonstrates the power and possibility to improve chemotherapeutic combinatorial therapeutics. The positive outcomes arising from this study suggest that lower eukaryotic systems, such as the fission yeast, will offer a cheaper and faster alternative to screen for drug combinations that are more likely to offer a better therapy. Fission yeast is also highly amendable to genetic manipulation, and thus useful for scaling up assay sizes to test many drugs and genes simultaneously.

In this work, we employed a serial dilution spotting assay, a commonly used method, which can be varied by streaking log-phase cells or by measuring changes in cell density^23^,^30^,^56^,^57^,^58^. Unlike previous studies, we have uniquely incorporated a computation of dose response, which, in our view, increases the accuracy of the assay. Furthermore, studying dose-response changes also helps to emphasize the pharmacological behavior of each compound over a range concentrations. However, the accuracy of cell growth can be further improved and incorporated into future experiments, such as the use of flow cytometric measurements of fluorescently labeled strains^59^ and the measurement of yeast colony sizes in conjunction with image-analysis software^60^.

One paradigm highlighted in this work is that cells possess a centralized, responsive network to counteract cytotoxic agents; this finding is consistent with a large-scale chemogenomic fitness screen in budding yeast, which uncovered a 45-gene cluster that coordinated the response of cells to small pharmacological molecules^42^. Mutants of the DXR genes showed differential sensitivity towards multiple DNA damaging agents, indicating an unequal contribution of each DXR gene toward this hypothesized centralized multidrug resistance (MDR) network; this unequal contribution may be partially explained by the differences in connectivity among partners in the network^61^,^62^ or specificity for the central target of the drugs, such as that demonstrated for topoisomerase II in response to doxorubicin^63^.

The clustering of drug hypersensitivity exhibited by the DXR genes suggests a hierarchical architecture in which smaller networks are integrated into progressively more extensive networks that are directed against an increasingly larger number of drugs. This model depicts that a targeted destabilization of widely separated sub-networks would result in stronger synergistic effects as compared to a disruption of closely related ones. Hence, a drug combination that can destabilize a more widely separated nexus within the MDR network would be expected to triumph as an effective combination. An understanding of the unique network architecture specific for cancer cells would help shed light on how drug resistance is coordinated in cancer cells, and this knowledge would facilitate the targeted killing of cancer cells over that of normal cells.

We recently showed that two DXR mutants, Δ*mcl1* and Δ*mhf1*, disrupt chromatin integrity at the fission yeast centromere, and that this activity could be suppressed by a point mutation in topoisomerase II (Top2) (*top2-191*)^63^. The Δ*mcl1* mutant showed sensitivity to four of the six (not MMS and SAHA) drugs tested, whereas Δ*mhf2* was sensitive to all but SAHA (Fig. 1b). This hypersensitivity toward multiple cytotoxic agents suggests that disruption to centromeric integrity may be a more widespread mechanism underlying susceptibility to cytotoxic chemotherapeutic agents besides doxorubicin. The disruption of centromeric integrity may also underlie the rampant hypersensitivity of mutants in DASH complex components to a DNA damaging agent that does not directly undermine microtubule stability^64^ (Figs 1b, 3).

Pmd1 is the fission yeast homolog of the human ATP-binding cassette (ABC) transporter permeability glycoprotein (P-gp/MDR1/ABCB1)^25^ and we have previously shown that Pmd1 acts synergistically with the vacuolar-ATPase pathway to govern doxorubicin hypersensitivity in human cervical carcinoma cells, probably via modulation of the intracellular accumulation of doxorubicin^25^. In humans, P-gp has been implicated in resistance against multiple drugs^4^ and it is surprising that Δ*pmd1* was among the mutants that showed only limited sensitivity towards the drugs tested^25^. This limited sensitivity may be due to peculiar substrate specificities or functional differences between the fission yeast Pmd1 and human P-gp. However, it is also possible that P-gp in human cells does not work alone but in conjunction with other membrane-associated transporters, resembling the functional cooperation between Pmd1/P-gp and V-ATPase in counteracting doxorubicin cytotoxicity^25^. In this context, it is possible that a network of different transporters may be involved in controlling drug resistance by regulating the levels of drug accumulation, which may be further modulated by the metabolism of these drugs within the cells.

A potential complication in using a cell-based assay to test drug cooperativity is the difference in bioavailability of the drugs due to the differences in transporter profiles among different cell types, which has been reported with different human cells and between *in vitro* cell culture models and *in vivo* animal models^65^,^66^. The results from cell-based models must therefore be interpreted with caution. Based on a similar extrapolation, the transporter expression profiles would also be different between the yeast systems and human cells. Hence additional experiments using whole animal testing and/or xenografts would be needed to validate the results from cell-based models. In this aspect, despite the perceived difference between yeast and human cultured cells, our previous results still suggest the value of the yeast system in elucidating plausible, cooperatively acting drug combinations^25^. Furthermore, screening yeast cells offers advantages of speed and cost efficiency over that in human cells, and provides the opportunity to target fewer drugs in a more specific manner.

Doxorubicin is widely employed in chemotherapeutic drug combinations along with cisplatin, for example, in the cells of urothelial cancers or with cisplatin, vinblastine and methotrexate in gastric cancer^51^,^65^. Here, we discovered the HDACi SAHA as a new partnering drug for the doxorubicin–cisplatin combination in gastric adenocarcinoma and cervical carcinoma cells. There is a recent resurgence in the utility of HDACis for drug sensitization. Initial studies emphasized the use of HDACis in the pretreatment of cancer cells to increase the cytotoxicity of the drugs, presumably by decondensing the chromatin for improved accessibility of other cytotoxic DNA-adduct-causing drugs^66^,^67^. However, here we show that SAHA exerts a similar effect to elicit an increase in the cytotoxicity of doxorubicin and further accentuates the effect of cisplatin. The cooperative effect of the three drugs was applied at a level where each separately did not show much effect (Fig. 4).

While we were preparing this manuscript, a phase I/II clinical trial was reported, demonstrating that the addition of belinostat, a HDACi, can improve a drug regimen containing cisplatin and doxorubicin (also with cyclophosphamide) in thymic epithelial tumors^68^. This report increases our confidence that drug combinations that target resistance can be determined from yeast synthetic lethal network analyses and provides evidence for the utility of SAHA in the triple combination. Future large-scale profiling of drugs and genetic interactions are expected to revolutionize the application of drug combination chemotherapy.

In conclusion, our work provides a novel approach for determining potential chemotherapeutic drug combinations for the sensitization of human cancer cells. Interfacing yeast synthetic lethality and human studies may provide a cheap and more targeted workflow with which to address drug resistance.

## Methods

### Drugs

Methyl methanesulfonate (MMS) and thiabendazole (TBZ) were purchased from Sigma-Aldrich (St Louis, MO). Camptothecin (CPT), hydroxyurea (HU), doxorubicin and cisplatin were from Wako Pure Chemical Industries Ltd (Osaka, Japan). Vorinostat/suberoylanilide hydroxamic acid (SAHA) was synthesized in-house (see Supplementary Materials)^69^. Doxorubicin was dissolved in water, SAHA (refer supplementary procedure) in DMSO, and cisplatin (Wako, Pure Chemical Industries, Ltd, Osaka, Japan) in 10% NaCl. All drugs were further diluted with their respective solvents prior to use according to manufacturers’ recommendations.

### Fission yeast techniques

A standard procedure for the treatment of fission yeast was followed^70^. Fission yeast strains were serially diluted and then spotted onto drug plates, as previously reported^24^,^25^. Cell growth was analyzed at two time points, at days 3 and 7, after spotting on the agar media. We chose these two time points in order to better estimate the effects of the drugs on cell growth as compared with cell viability. Specifically, during the growing phase and before maximum growth is attained (on day 3), cells will exhibit growth retardation if both their growth and viability are affected. However, by day 7 and when the strains have reached maximum growth, only cells that have lost viability in response to the drug will exhibit growth defects. Cells that show reduced growth but no loss in viability will show growth that is near or equivalent to that of WT cells. All DXR strains were obtained in a previous screen that tested for the hypersensitivity of fission yeast single-gene knockout strains from commercial libraries (versions 1.0 and 2.0) (Bioneer, Daejeon, Korea) and subsequently converted into the prototrophic genetic background by backcrossing with prototrophic WT strains.

### Calculation of sensitivity score (s-score)

The step-wise derivation of the s-score is described in Supplementary Fig. 7. Growth fitness of the strains was obtained by comparing the growth of strains on drug-treated plates relative to that on non-drug-treated plates. The fitness values obtained for mutants were normalized against that of the WT cells before a logarithmic transformation was performed. The s-score was obtained from the mean of the sum of the relative fitness measurement resultant values obtained.

### Interaction network construction tool

The online network construction tool, String version 9.1^36^, was employed to discover interaction links among the previously identified DXR genes.

### Human cell culture and drug treatment

Human gastric adenocarcinoma (AGS) cells (ATCC, Manassas, VA) were grown in RPMI media (Sigma-Aldrich) supplemented with 10% fetal bovine serum (FBS) (Life Technologies), whereas human cervical carcinoma (HeLa) (ATCC) and human embryonic kidney cells (HEK293) (ATCC) were maintained in EMEM media (Sigma-Aldrich) supplemented with 10% FBS. Drug concentrations were determined by dose-response assay, where cells were seeded into 96-well culture plates and treated in triplicate with various drug concentrations of doxorubicin: (0, 0.001, 0.01, 0.1, 1, 5, 10, 25, 100 μM) and SAHA (0, 0.39, 0.78, 1.56, 3.13, 6.25, 12.5, 25, 50 μM). The doxorubicin and SAHA concentrations that elicited approximately 30% cell death were selected for cytotoxicity tests, in which the cells were then exposed to varying concentrations of cisplatin (0, 1.65, 3.125, 6.25, 12.5, 25, 50, 100 μM; Fig. 4). Cell viability was determined with cck-8 assay, according to the manufacturer’s protocol (Dojindo, Kumamoto, Japan). Triplicates of each treatment were performed over five separate sets of experiments.

### Statistical analyses

Dose-response curves of the following treatments were plotted with standard deviations: varying cisplatin only; 5 μM SAHA with varying cisplatin; 5 μM doxorubicin with varying cisplatin; and the triple treatment of 5 μM SAHA with 5 μM doxorubicin in varying cisplatin. Cell viability at each concentration of cisplatin was compared against the 0 μM cisplatin for the respective treatment group. The statistical significance of the synergistic effects was examined with a two-tailed Student’s *t*-test and performed using Microsoft Excel (2007). A simulation was performed to ascertain similarity between CPT, HU and MMS, as previously reported^41^. For each pair of drugs *x* and *y*, two sets (*S*_*x*_ and *S*_*y*_) each of 10,000 random profiles were generated. The significance of the similarity *s*(*x, y*) between *x* and *y* was assessed by the comparison of *s*(*x, y*) with the correlation between two random profiles taken respectively from *S*_*x*_ and *S*_*y*_.

## Additional Information

**How to cite this article**: Nguyen, T. T. T. *et al.* Predicting chemotherapeutic drug combinations through gene network profiling. *Sci. Rep.* **6**, 18658; doi: 10.1038/srep18658 (2016).

## Supplementary Material

Supplementary Information
