# Supplementary material for: Predicting chemotherapeutic drug combinations through gene network profiling
Source: Sci Rep. 2016 Jan 21;6:18658. doi: 10.1038/srep18658 (PMC4726371; doi:10.1038/srep18658)
Supplement: Supplementary Information [file srep18658-s1.pdf]

Supplementary Information

(11 Figures and 1 supplementary method)

Predicting chemotherapeutic drug combinations through gene network profiling

Thi Thuy Trang Nguyen[1,2], Jacqueline Kia Kee Chua[1,8], Kwi Shan Seah[1,2], Seok Hwee Koo[5,10], Jie Yin Yee[2,5], Eugene Guorong Yang[6], Kim Kiat Lim[1,2], Shermaine Yu Wen Pang[1], Audrey Yuen[9], Louxin Zhang[7], Wee Han Ang[4,8], Brian Dymock[6], Edmund Jon Deoon Lee[2,5] and Ee Sin Chen[1,2,3,4,*]

[1]Department of Biochemistry, National University of Singapore, Singapore, [2]National University Health System (NUHS), Singapore, [3]Synthetic Biology Research Consortium, National University of Singapore, [4]NUS Graduate School for Integrative Sciences and Engineering, [5]Department of Pharmacology, [6]Department of Pharmacy, [7]Department of Mathematics, [8]Department of Chemistry, Faculty of Science, National University of Singapore, Singapore, [9]School of Chemical and Life Sciences, Singapore Polytechnic, Singapore,[10]Changi General Hospital, Ministry of Health, Singapore

*Corresponding author Email: bchces@nus.edu.sg

Supplementary Figures and Supplementary Method

Supplementary Figure 1 Spotting of doxorubicin resistance (DXR) strains obtained with indicated concentrations of hydroxyurea (HU).

Supplementary Figure 2 Spotting of doxorubicin resistance (DXR) strains obtained with indicated concentrations of methyl methanesulfonate (MMS).

Supplementary Figure 3 Spotting of doxorubicin resistance (DXR) strains obtained with indicated concentrations of camptothecin (CPT).

Supplementary Figure 4 Spotting of doxorubicin resistance (DXR) strains obtained with indicated concentrations of thiabendazole (TBZ).

Supplementary Figure 5 Spotting of doxorubicin resistance (DXR) strains obtained with indicated concentrations of cisplatin.

Supplementary Figure 6 Spotting of doxorubicin resistance (DXR) strains obtained with indicated concentration of suberoylanilide hydroxamic acid (SAHA).

Supplementary Figure 7 Derivation of sensitivity scores (s-scores) from cell growth phenotypes grown on a series of drug concentrations. (**a**) Quantitation of the fold sensitivity from the 10-fold serially diluted drug plates. (**b**) Formula expressing the logarithmic transformation of the mean fold sensitivity relative to the growth of cells on non-drug-treated plates. (**c**) Schematic representation of the interpretation of the s-score values. A value of 0 is taken as no sensitivity, a negative s-score represents hypersensitivity, and a positive value means resistance.

Supplementary Figure 8 Serial dilution of wild-type (WT) prototrophic fission yeast strains of mating type h^+ (975) and h^- (972) on 0, 15, 30, 75 µg/ml doxorubicin alone or in conjunction with 0.01% methyl methanesulfonate (MMS), 8 µg/ml thiabendazole (TBZ), 2

mM hydroxyurea (HU), 8 µM camptothecin (CPT), 1.2 mM cisplatin, 5 mM suberoylanilide hydroxamic acid (SAHA) or with 1.2 mM cisplatin and 5 mM SAHA. These concentrations were selected as they did not significantly reduce WT cell viability. Cell growth was assessed on days 3 and 7 after spotting onto drug plates.

Supplementary Figure 9 DXR mutants exhibit hypersensitivity towards the triple combination of cisplatin, SAHA and doxorubicin. WT, *Δrhp51*, *Δrav1*, *Δvps35*, *Δcaf1* and *Δtim11* were 10-fold serially diluted and spotted onto media containing 0, 15, 30, 75 µg/ml doxorubicin alone or in combination with 1.2 mM cisplatin, 5 mM suberoylanilide hydroxamic acid (SAHA). Cell growth was analyzed on days 3 and 7 after drug treatment.

Supplementary Figure 10 Sensitization of human cervical carcinoma (HeLa) cells to doxorubicin via concurrent treatment with cisplatin and suberoylanilide hydroxamic acid (SAHA). (**a**) Cells were co-treated with varying concentrations of cisplatin (halving dilutions from 100 µM to 0) in the presence of 10, 15, or 25 µM SAHA, or 0.1, 1 or 5 µM doxorubicin or with a triple combination (10 µM SAHA constant). (**b**) Dose response effect on the viability of HeLa cells was analyzed following treatment with varying concentrations of cisplatin alone (blue), in combination with 0.1 µM doxorubicin (green) or 10 µM SAHA (red), or 0.1 µM doxorubicin and 5 µM SAHA (purple). (**c**) Similar to (**b**) except 1 µM doxorubicin was used.

Supplementary Figure 11 Human non-cancerous embryonic kidney (HEK293) cells did not show cumulative cell killing in triple combination of doxorubicin, cisplatin and suberoylanilide hydroxamic acid (SAHA). (**a**) Cells were co-treated with varying concentrations of cisplatin (halving dilutions from 100 µM to 0) in the presence of 5, 50, 100 µM SAHA, or 0.06, 0.15 or 1 µM doxorubicin or with a triple combination (5 µM SAHA constant). (**b**) Dose response effect on viability of HEK293 cells were analyzed after treating

with varying concentrations of cisplatin alone (blue), in combination with 0.1 µM doxorubicin (green) or 10 µM SAHA (red), or 0.06 µM doxorubicin and 5 µM SAHA (purple). (**c**) Similar to (**b**) except 0.15 µM doxorubicin was used.

Supplementary Materials and Methods Chemical synthesis of SAHA

Supplementary Figure 1

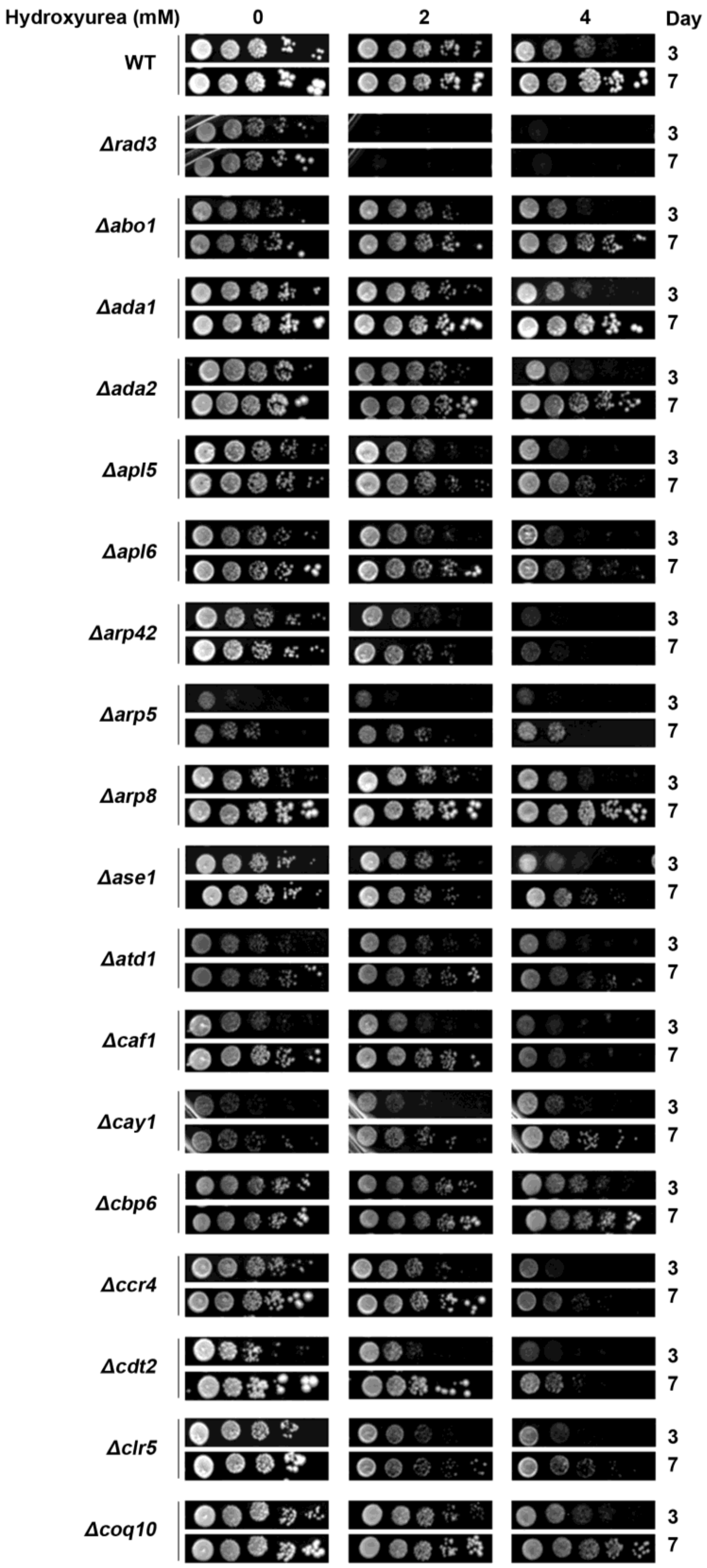

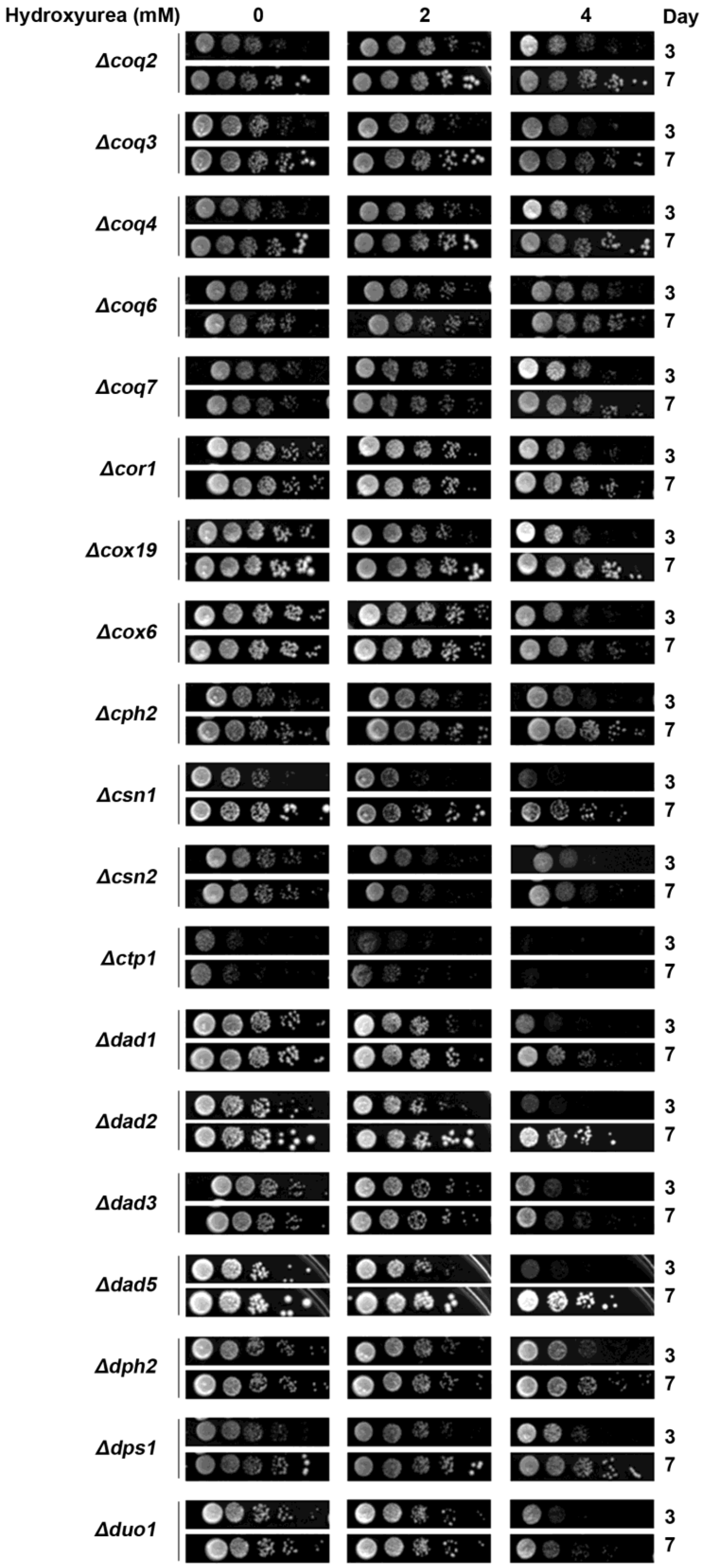
Hydroxyurea (mM)
0
2
4
Day
Δcoq2
Δcoq3
Δcoq4
Δcoq6
Δcoq7
Δcor1
Δcox19
Δcox6
Δcph2
Δcsn1
Δcsn2
Δctp1
Δdad1
Δdad2
Δdad3
Δdad5
Δdph2
Δdps1
Δduo1
3
7

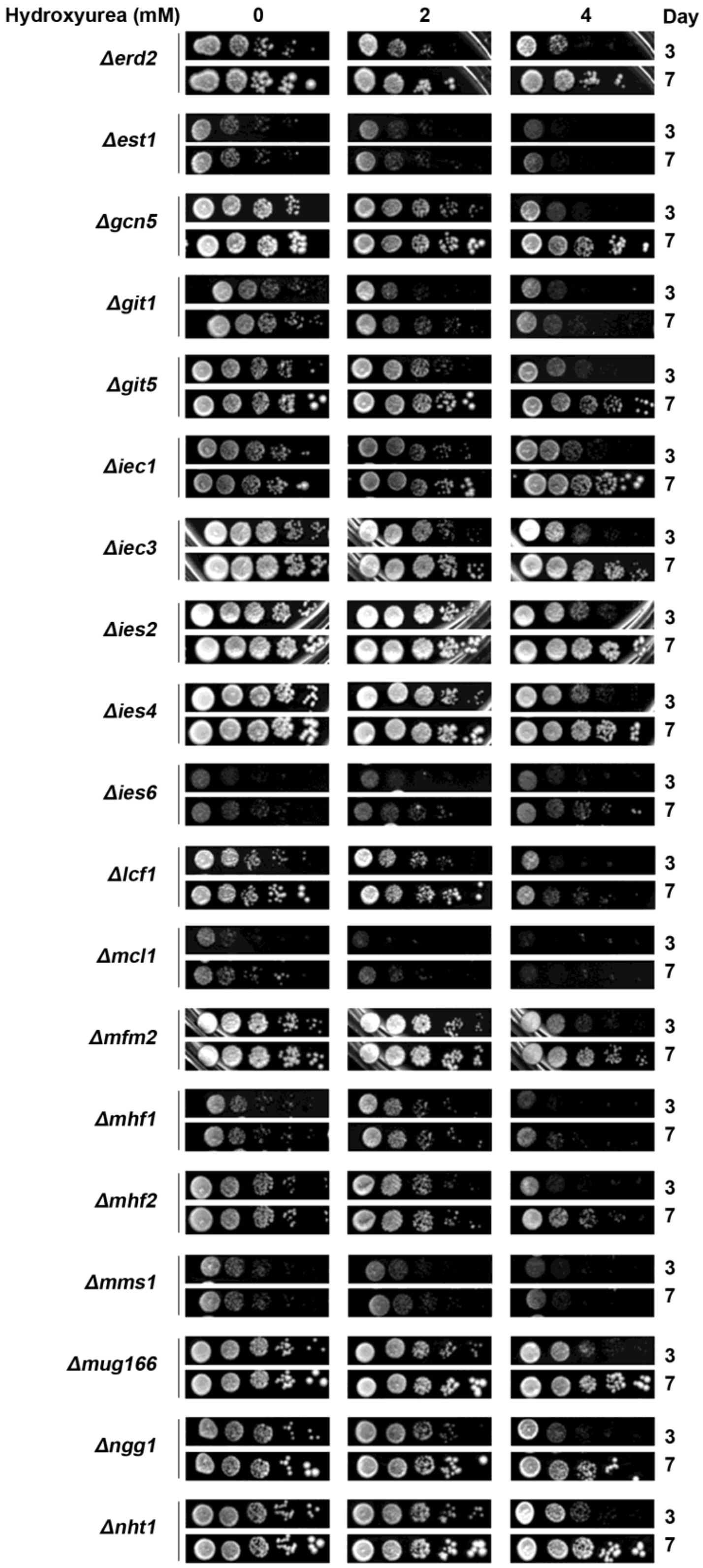

Hydroxyurea (mM)
0
2
4
Day
Δerd2
Δest1
Δgcn5
Δgit1
Δgit5
Δiec1
Δiec3
Δies2
Δies4
Δies6
Δlcf1
Δmcl1
Δmfm2
Δmhf1
Δmhf2
Δmms1
Δmug166
Δngg1
Δnht1
3
7

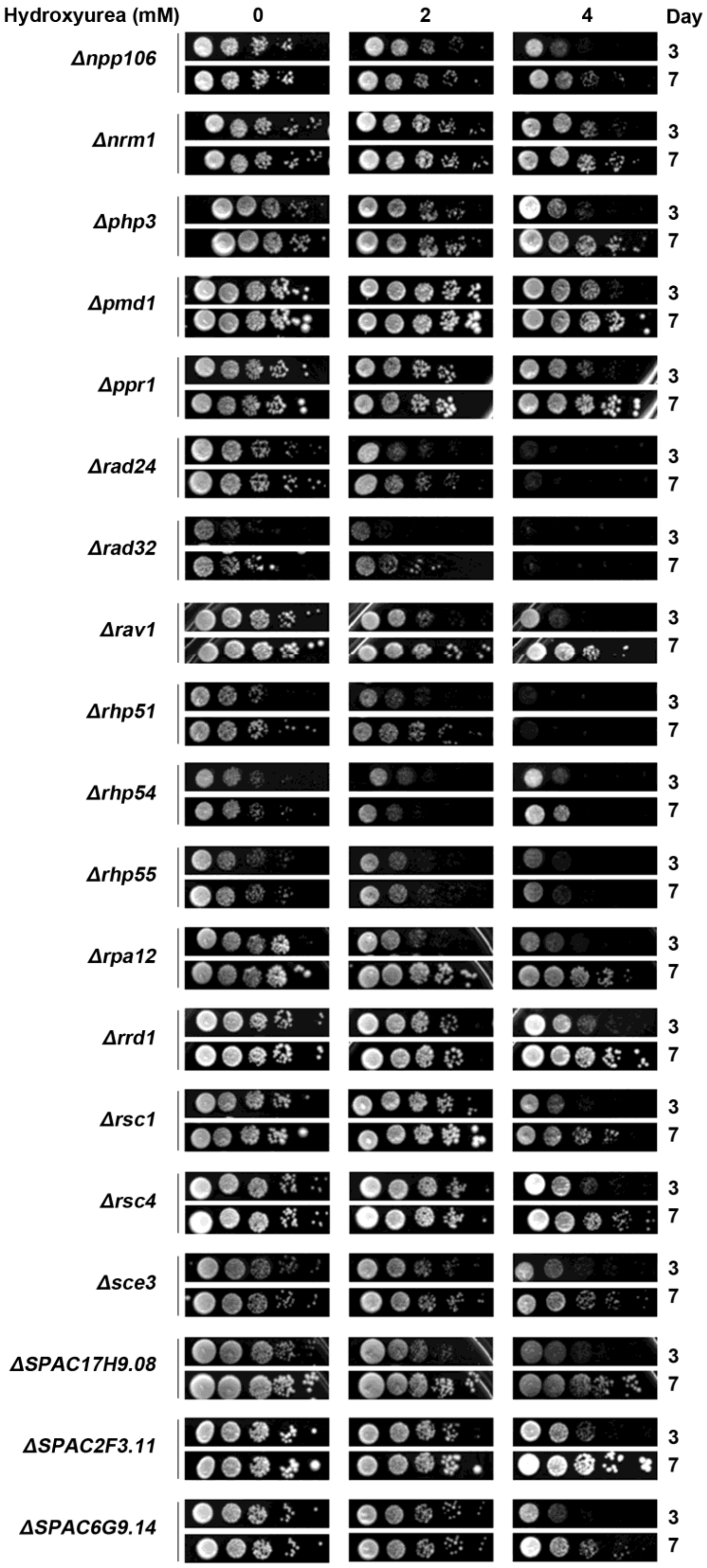

Hydroxyurea (mM)
0
2
4
Day
Δnpp106
Δnrm1
Δphp3
Δpmd1
Δppr1
Δrad24
Δrad32
Δrav1
Δrhp51
Δrhp54
Δrhp55
Δrpa12
Δrrd1
Δrsc1
Δrsc4
Δsce3
ΔSPAC17H9.08
ΔSPAC2F3.11
ΔSPAC6G9.14
3
7

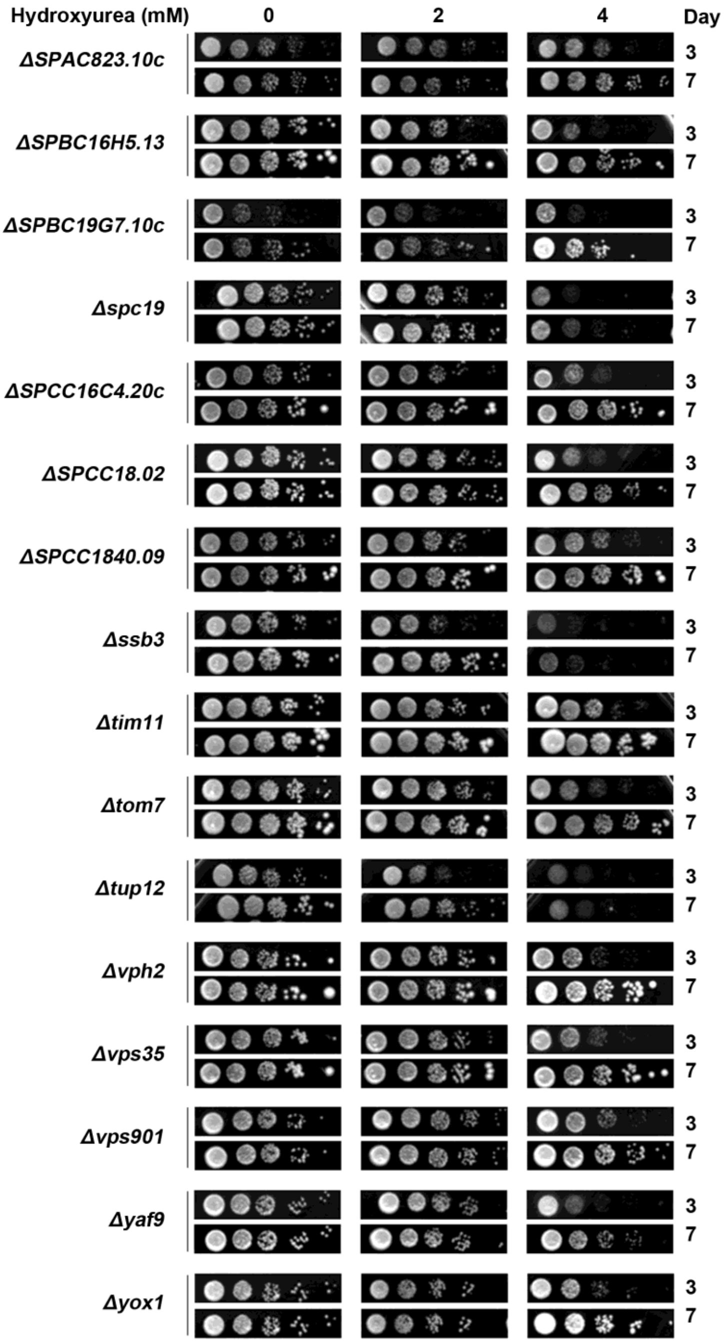
Hydroxyurea (mM)
0
2
4
Day
ΔSPAC823.10c
ΔSPBC16H5.13
ΔSPBC19G7.10c
Δspc19
ΔSPCC16C4.20c
ΔSPCC18.02
ΔSPCC1840.09
Δssb3
Δtim11
Δtom7
Δtup12
Δvph2
Δvps35
Δvps901
Δyaf9
Δyox1
3
7

Supplementary Figure 2

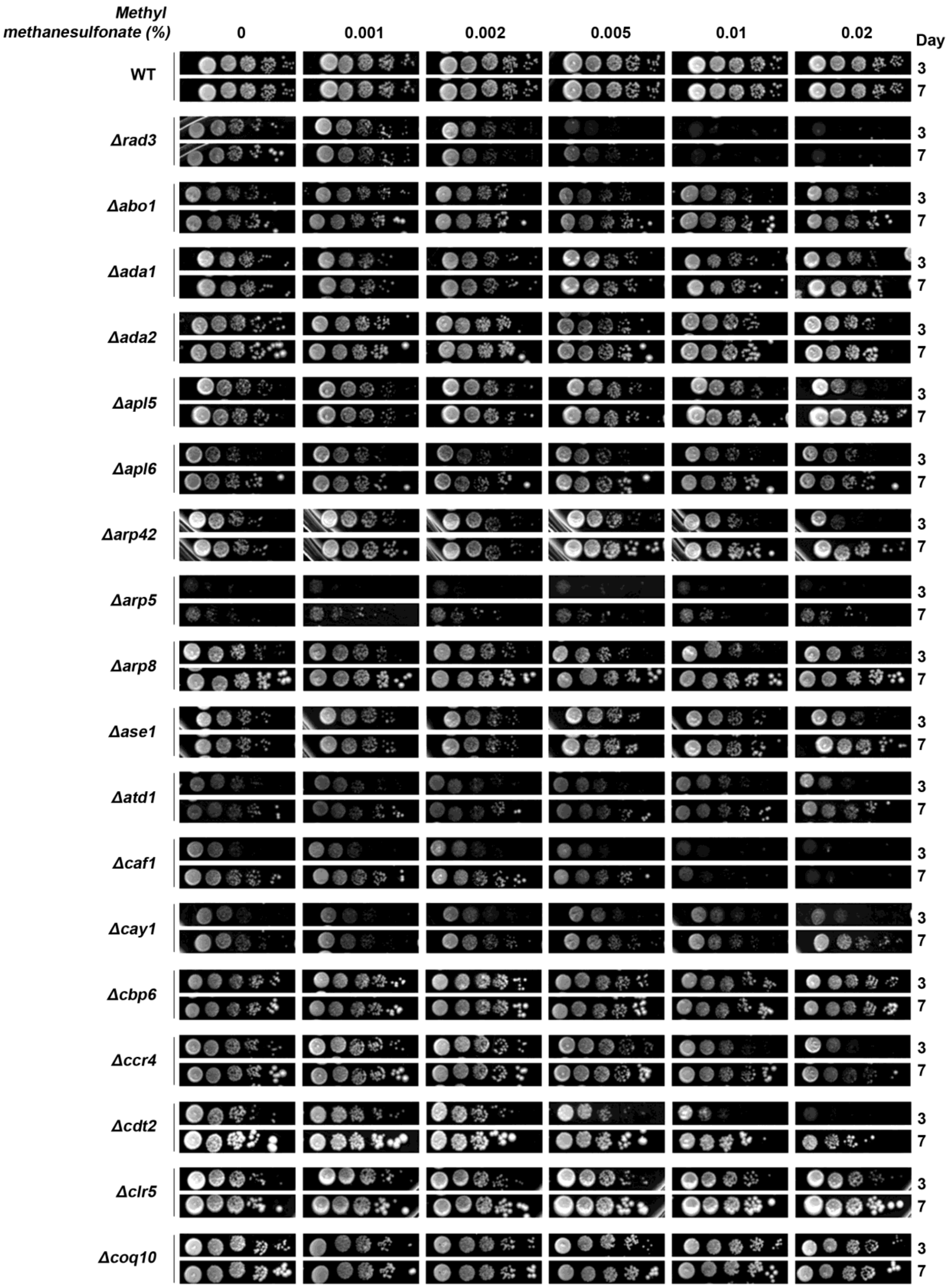

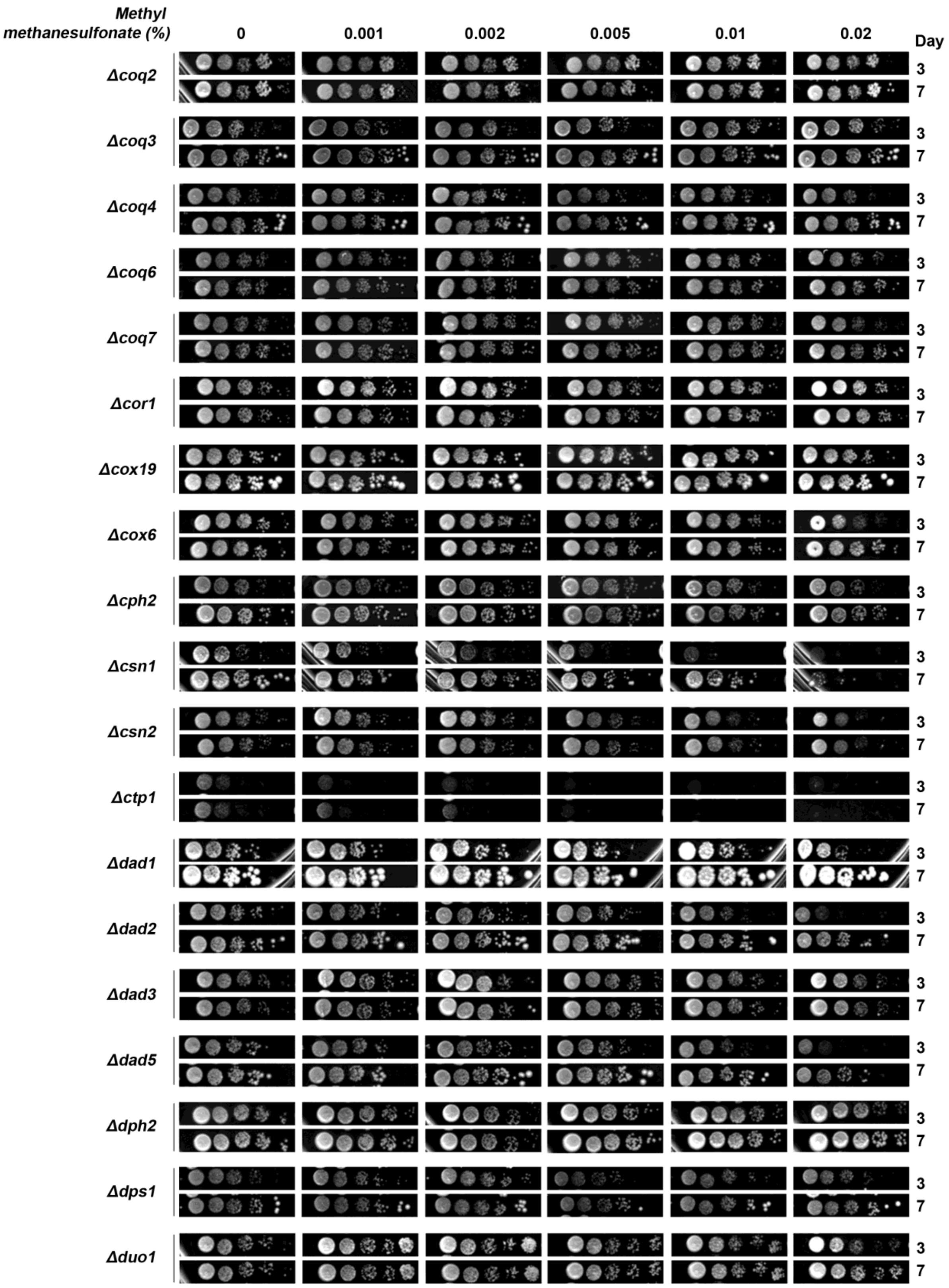
Methyl
methanesulfonate (%)
0
0.001
0.002
0.005
0.01
0.02
Day
Δcoq2
3
7
Δcoq3
3
7
Δcoq4
3
7
Δcoq6
3
7
Δcoq7
3
7
Δcor1
3
7
Δcox19
3
7
Δcox6
3
7
Δcph2
3
7
Δcsn1
3
7
Δcsn2
3
7
Δctp1
3
7
Δdad1
3
7
Δdad2
3
7
Δdad3
3
7
Δdad5
3
7
Δdph2
3
7
Δdps1
3
7
Δduo1
3
7

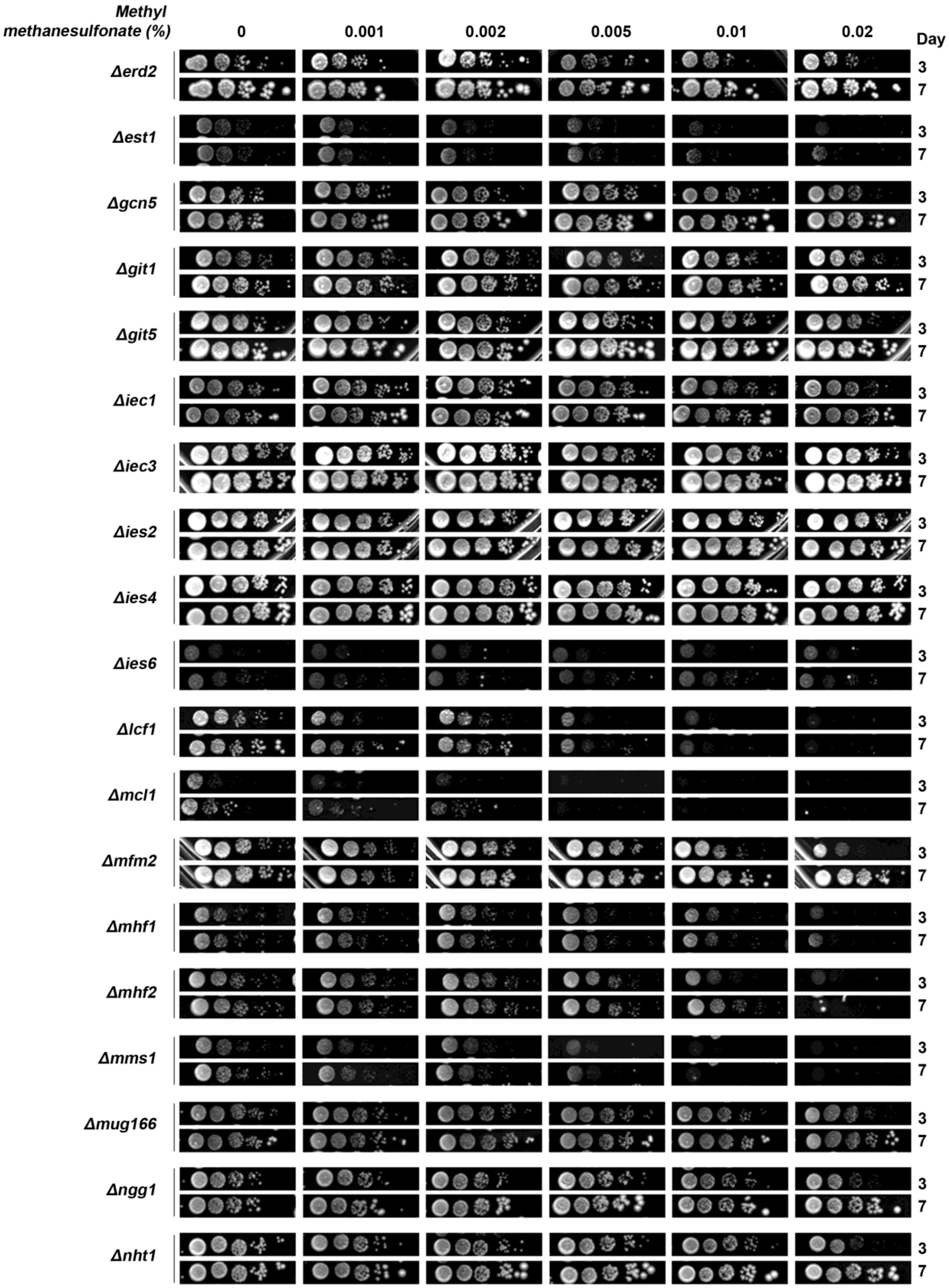
Methyl methanesulfonate (%)
0
0.001
0.002
0.005
0.01
0.02
Day
Δerd2
Δest1
Δgcn5
Δgit1
Δgit5
Δiec1
Δiec3
Δies2
Δies4
Δies6
Δlcf1
Δmcl1
Δmfm2
Δmhf1
Δmhf2
Δmms1
Δmug166
Δngg1
Δnht1
3
7

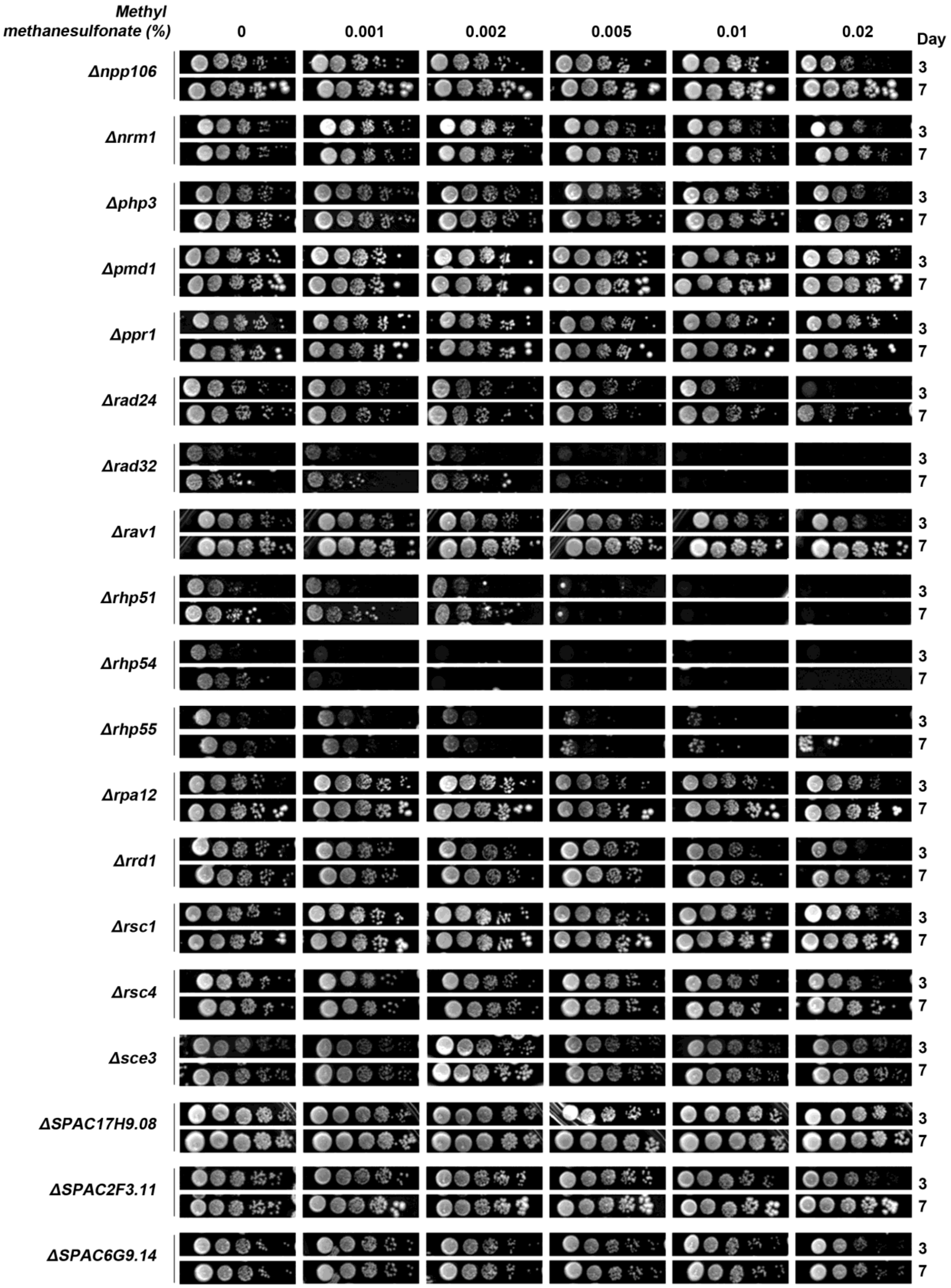

Methyl methanesulfonate (%)
0
0.001
0.002
0.005
0.01
0.02
Day
3
7
Δnpp106
Δnrm1
Δphp3
Δpmd1
Δppr1
Δrad24
Δrad32
Δrav1
Δrhp51
Δrhp54
Δrhp55
Δrpa12
Δrrd1
Δrsc1
Δrsc4
Δsce3
ΔSPAC17H9.08
ΔSPAC2F3.11
ΔSPAC6G9.14

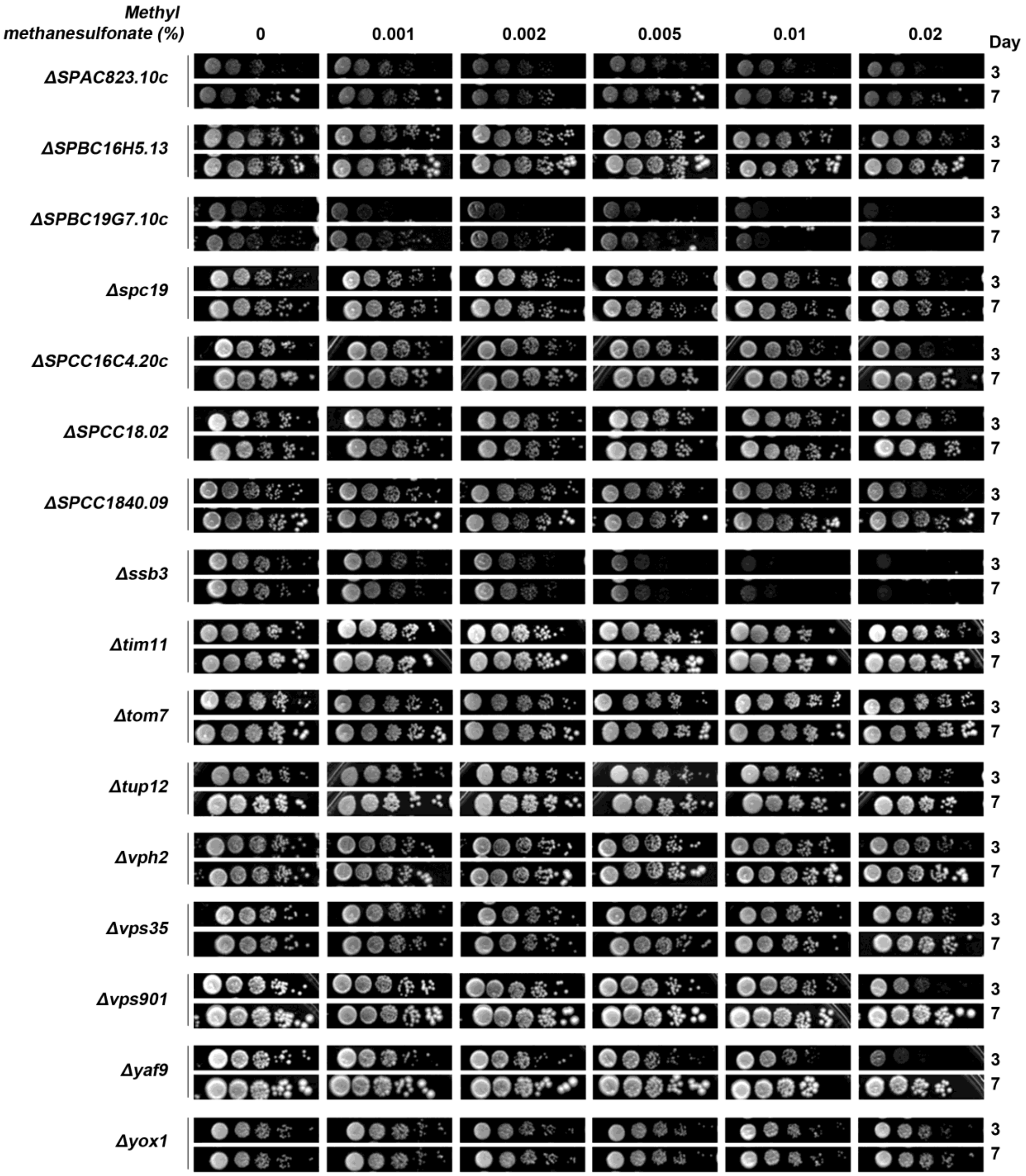

Methyl methanesulfonate (%)
0
0.001
0.002
0.005
0.01
0.02
Day
ΔSPAC823.10c
ΔSPBC16H5.13
ΔSPBC19G7.10c
Δspc19
ΔSPCC16C4.20c
ΔSPCC18.02
ΔSPCC1840.09
Δssb3
Δtim11
Δtom7
Δtup12
Δvph2
Δvps35
Δvps901
Δyaf9
Δyox1
3
7

Supplementary Figure 3

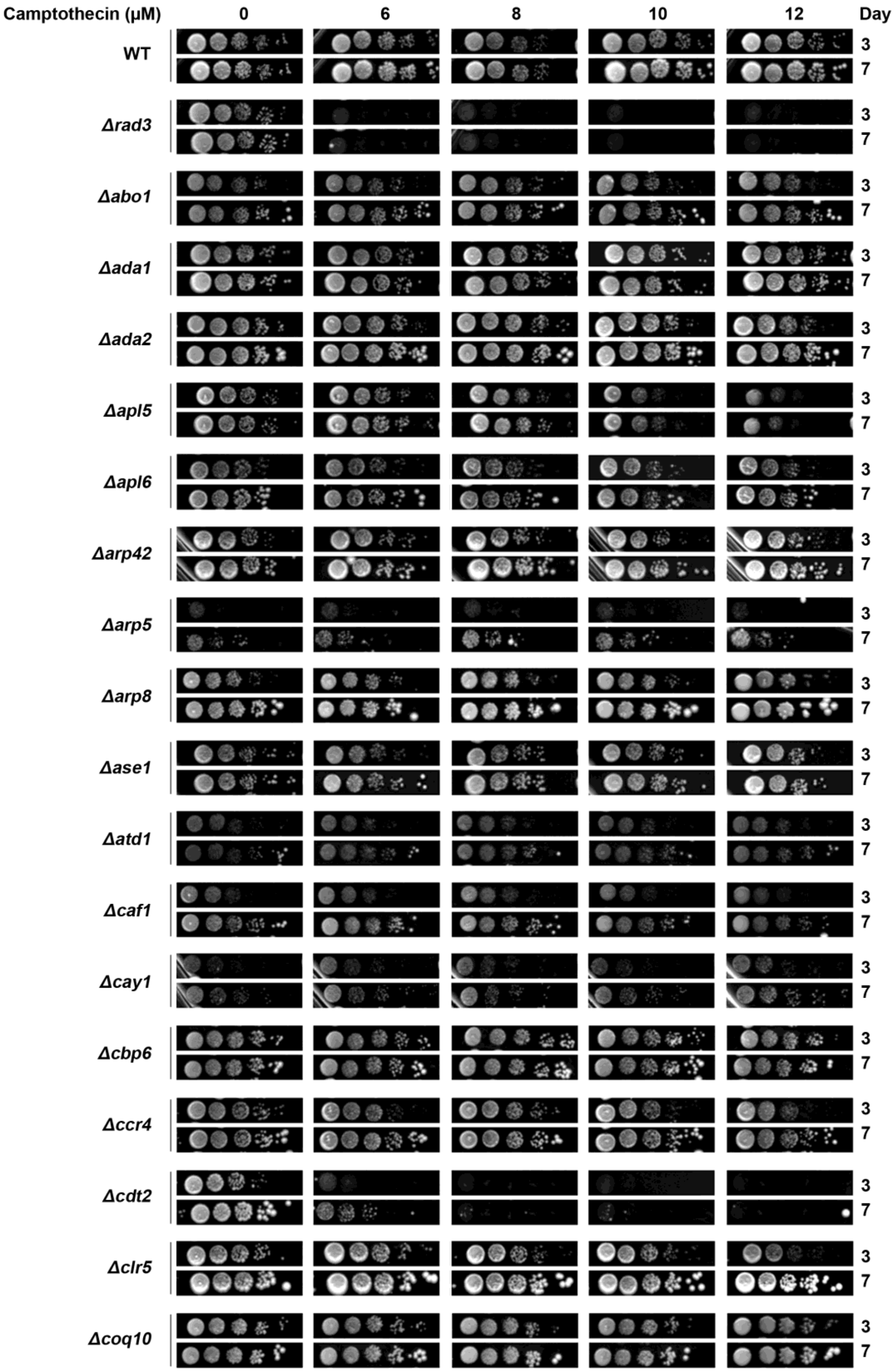

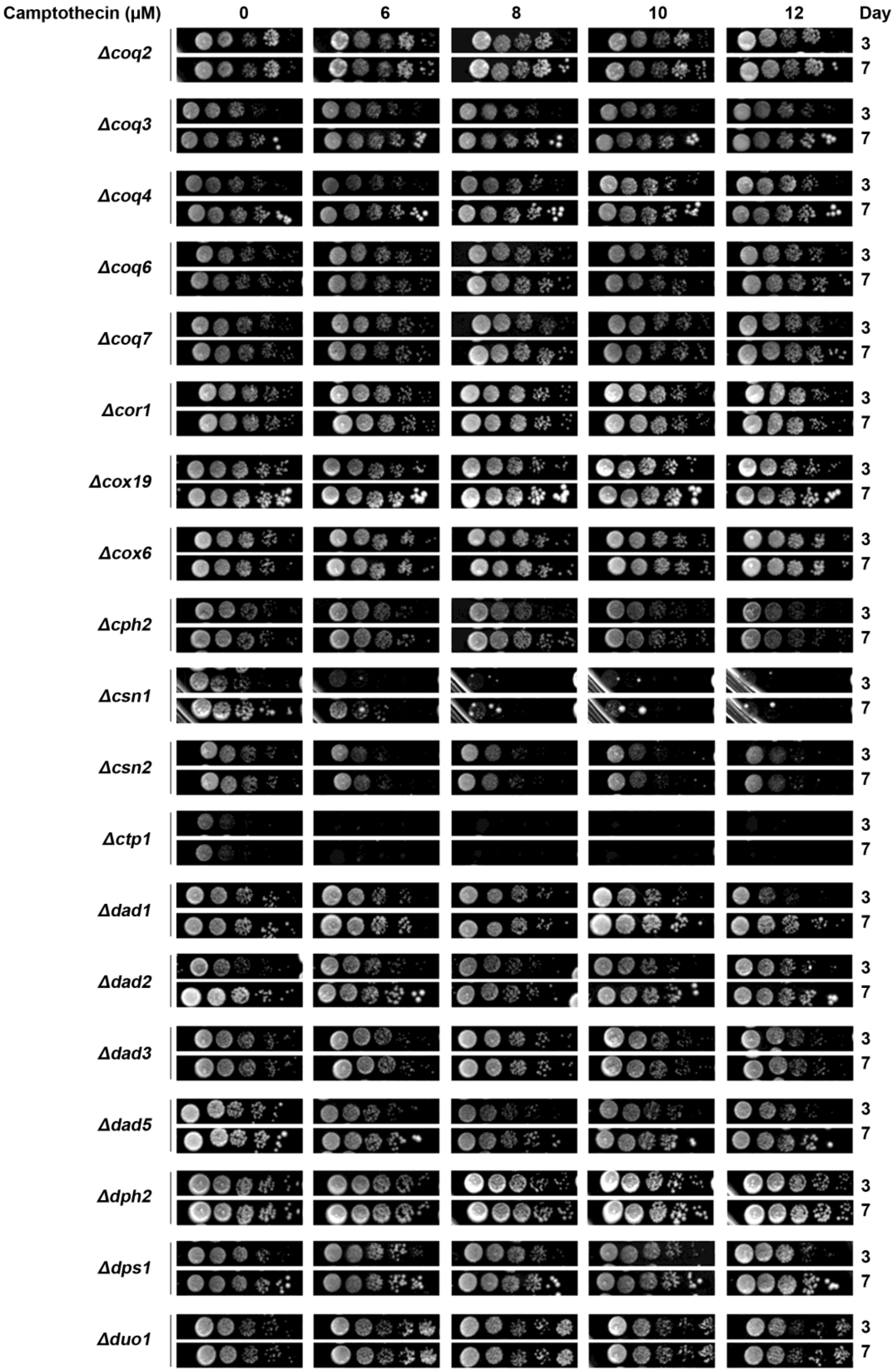
Camptothecin (μM)
0
6
8
10
12
Day
Δcoq2
Δcoq3
Δcoq4
Δcoq6
Δcoq7
Δcor1
Δcox19
Δcox6
Δcph2
Δcsn1
Δcsn2
Δctp1
Δdad1
Δdad2
Δdad3
Δdad5
Δdph2
Δdps1
Δduo1
3
7

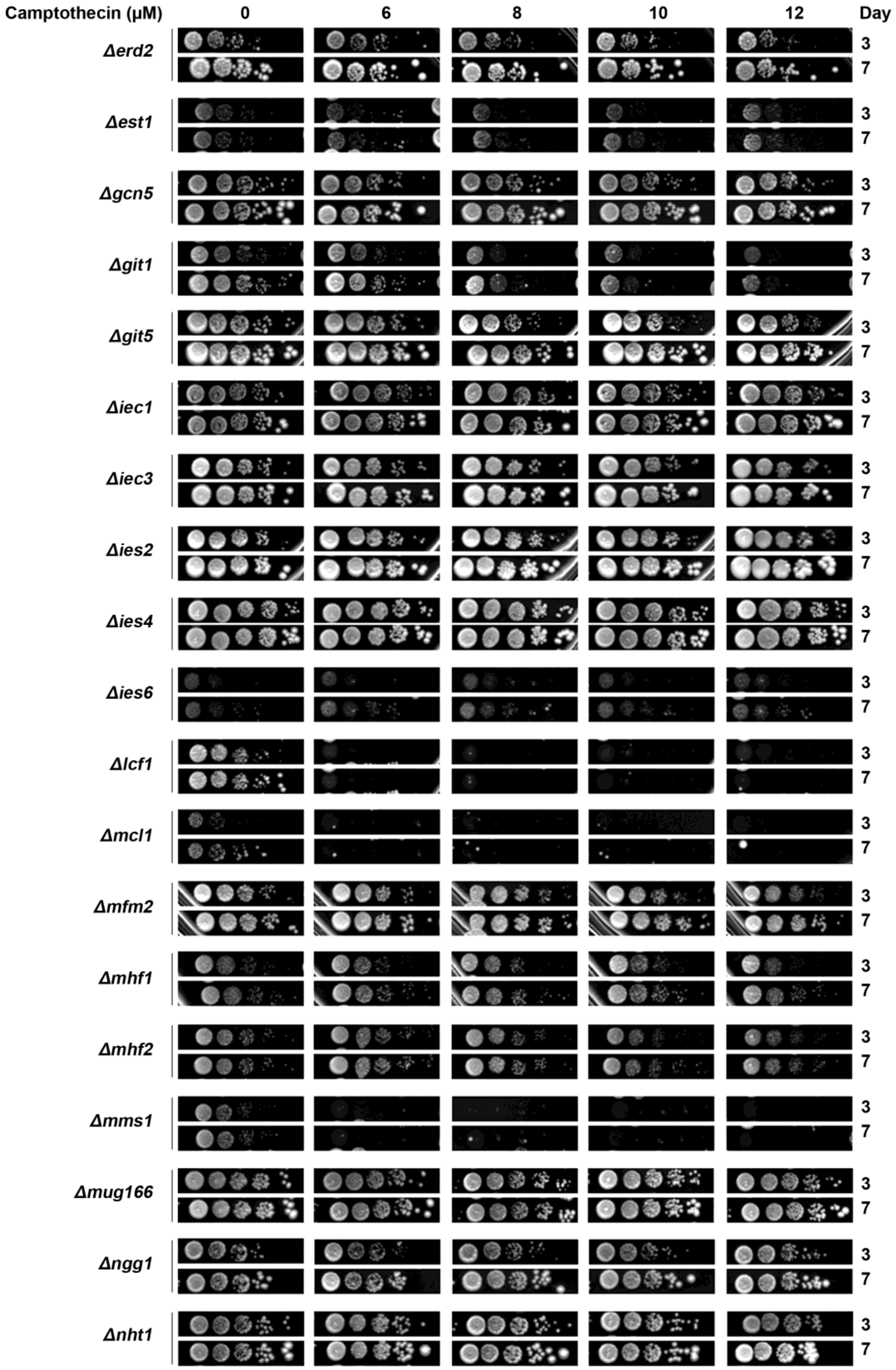
Camptothecin (μM)
0
6
8
10
12
Day
Δerd2
Δest1
Δgcn5
Δgit1
Δgit5
Δiec1
Δiec3
Δies2
Δies4
Δies6
Δlcf1
Δmcl1
Δmfm2
Δmhf1
Δmhf2
Δmms1
Δmug166
Δngg1
Δnht1
3
7

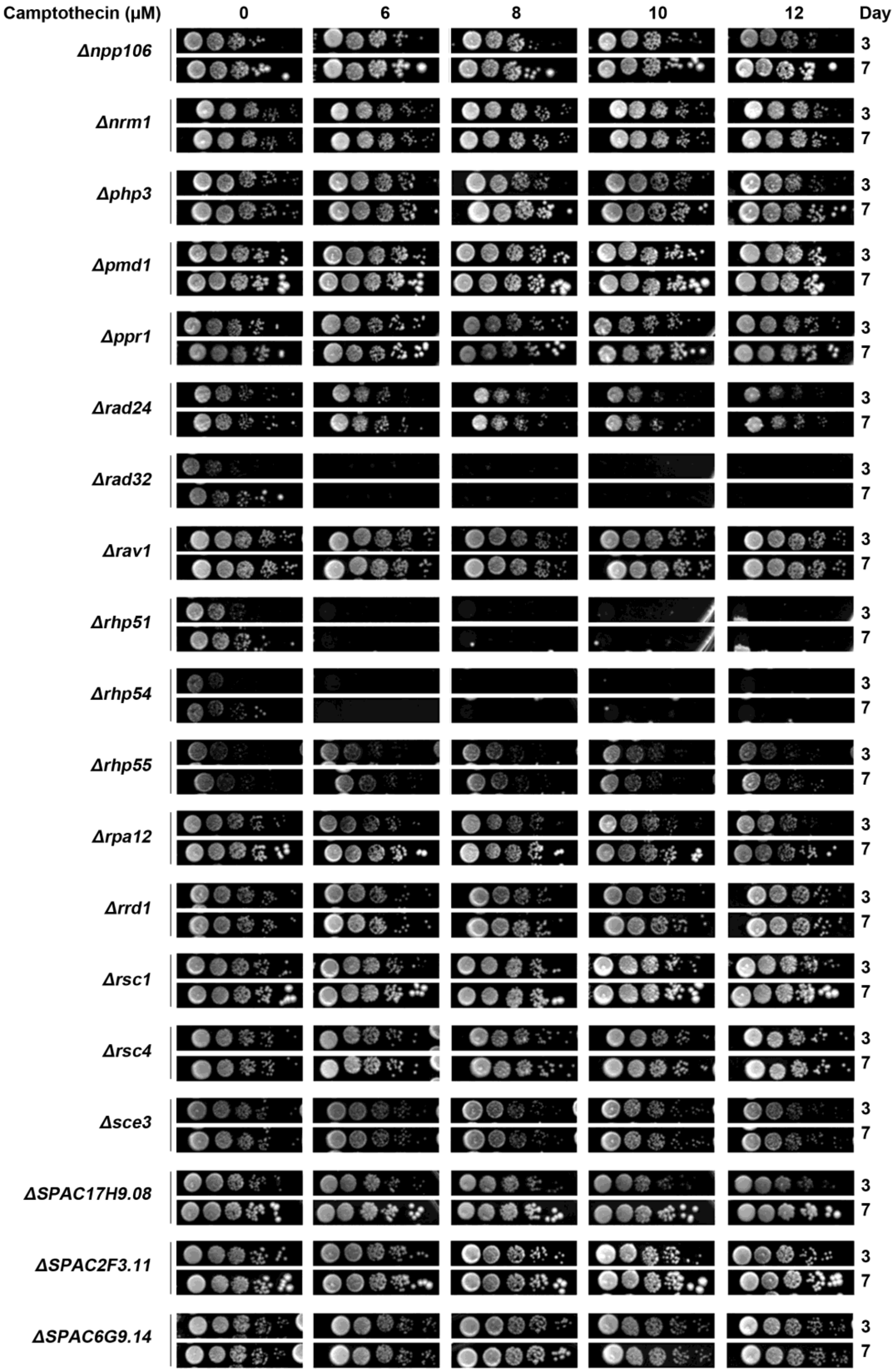

Camptothecin (μM)
0
6
8
10
12
Day
Δnpp106
Δnrm1
Δphp3
Δpmd1
Δppr1
Δrad24
Δrad32
Δrav1
Δrhp51
Δrhp54
Δrhp55
Δrpa12
Δrrd1
Δrsc1
Δrsc4
Δsce3
ΔSPAC17H9.08
ΔSPAC2F3.11
ΔSPAC6G9.14
3
7

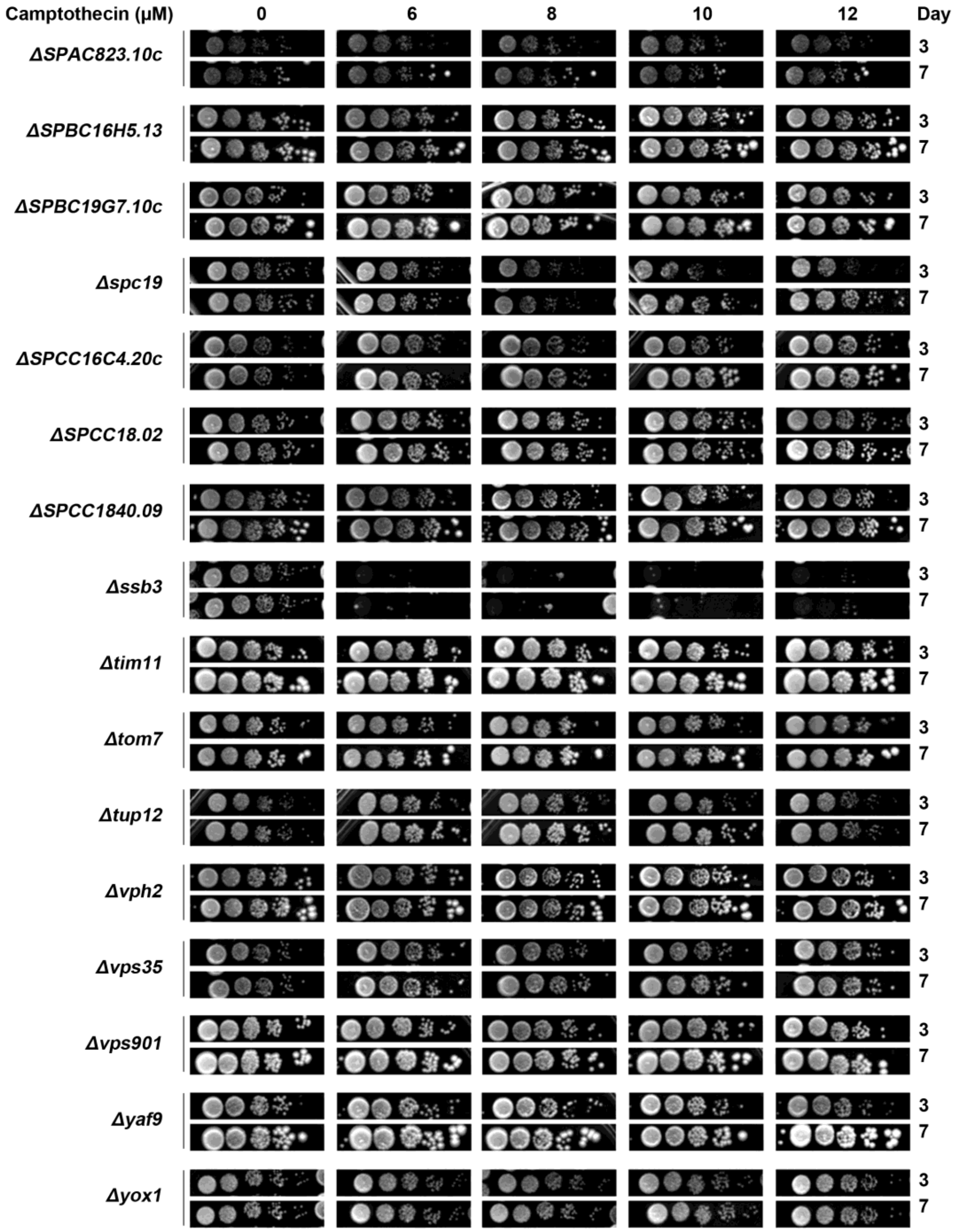
Camptothecin (μM)
0
6
8
10
12
Day
ΔSPAC823.10c
ΔSPBC16H5.13
ΔSPBC19G7.10c
Δspc19
ΔSPCC16C4.20c
ΔSPCC18.02
ΔSPCC1840.09
Δssb3
Δtim11
Δtom7
Δtup12
Δvph2
Δvps35
Δvps901
Δyaf9
Δyox1
3
7

Supplementary Figure 4

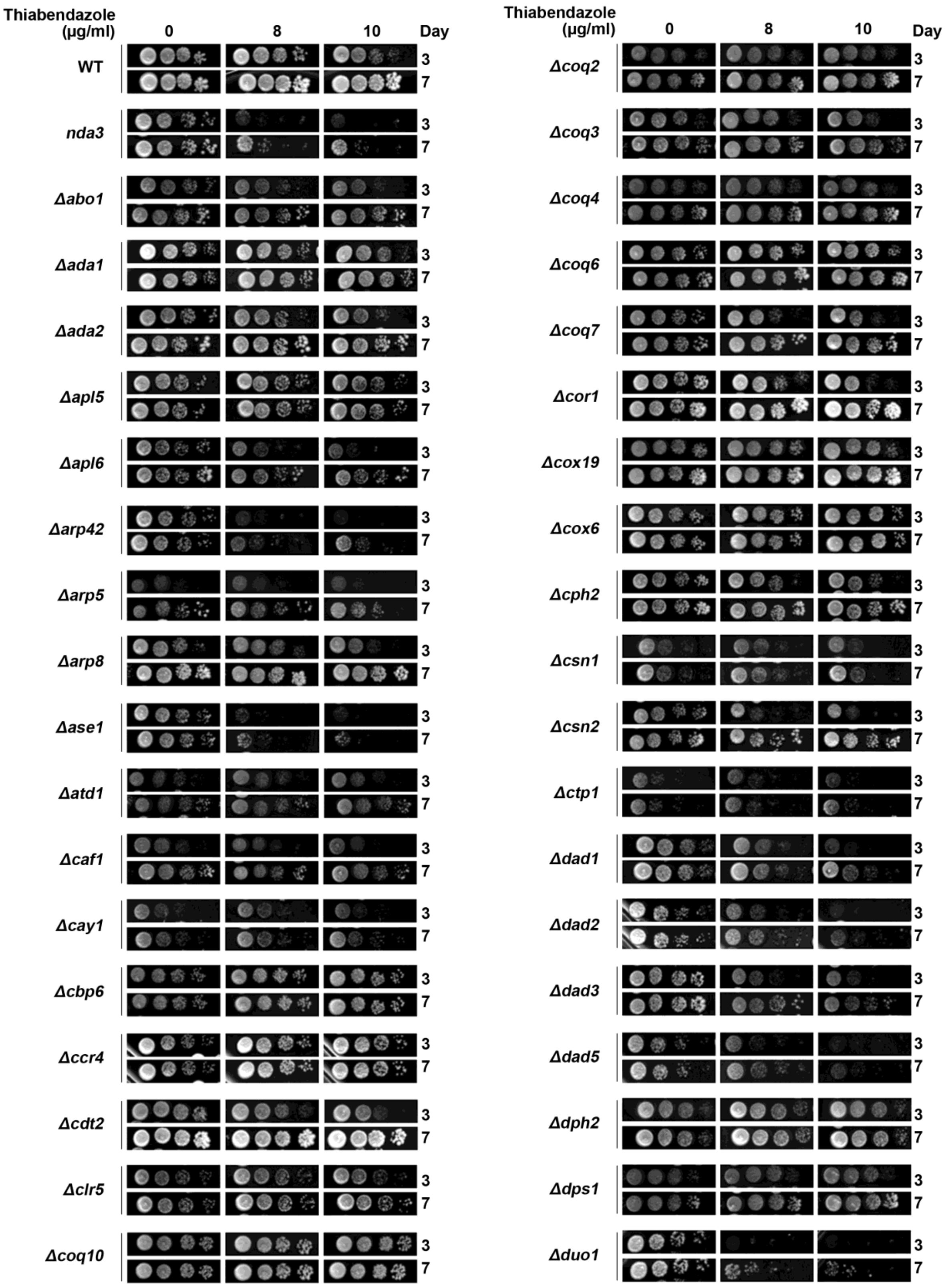

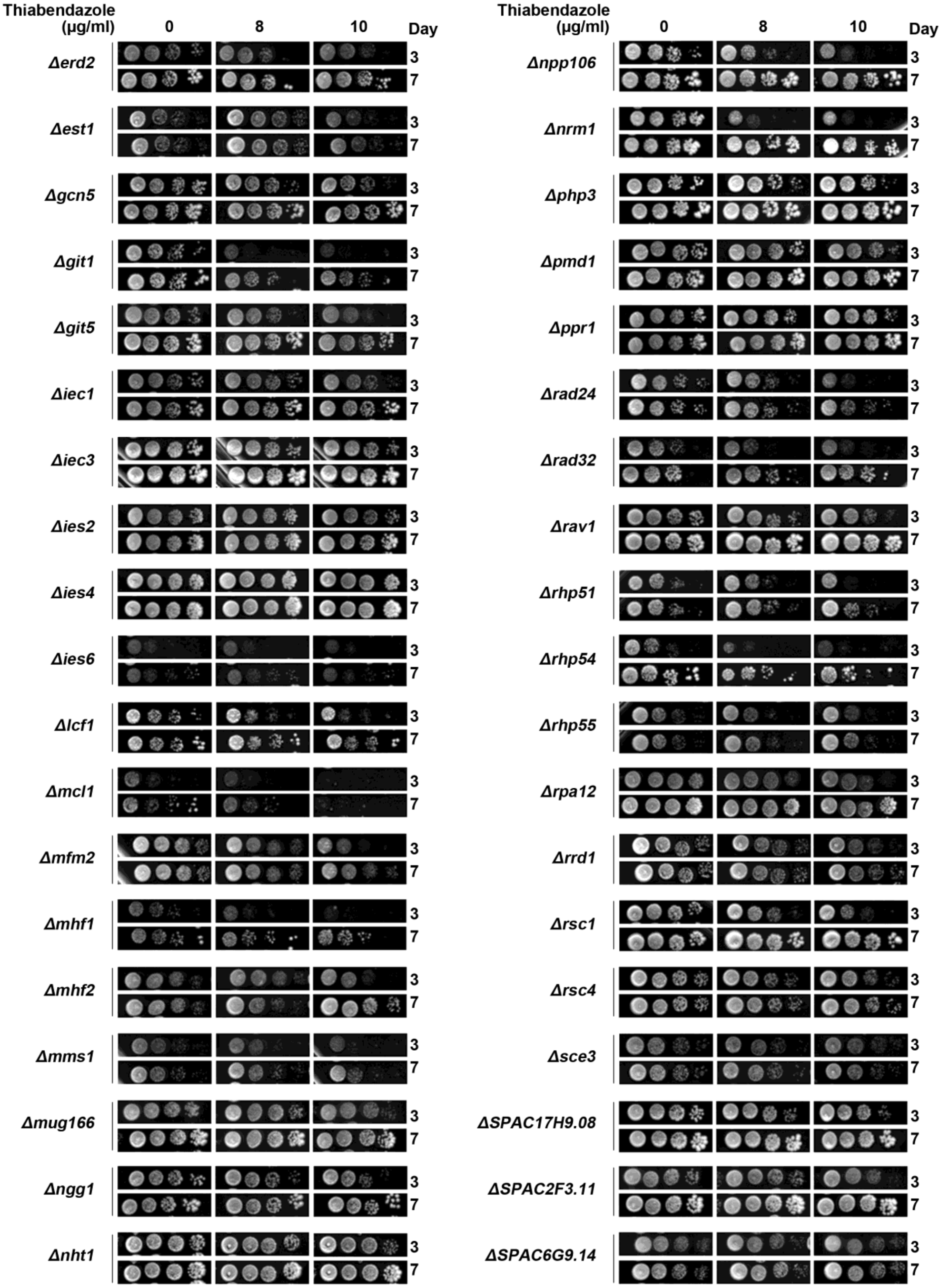

Thiabendazole (μg/ml)
0
8
10
Day
Δerd2
Δest1
Δgcn5
Δgit1
Δgit5
Δiec1
Δiec3
Δies2
Δies4
Δies6
Δlcf1
Δmcl1
Δmfm2
Δmhf1
Δmhf2
Δmms1
Δmug166
Δngg1
Δnht1
Δnpp106
Δnrm1
Δphp3
Δpmd1
Δppr1
Δrad24
Δrad32
Δrav1
Δrhp51
Δrhp54
Δrhp55
Δrpa12
Δrrd1
Δrsc1
Δrsc4
Δsce3
ΔSPAC17H9.08
ΔSPAC2F3.11
ΔSPAC6G9.14
3
7

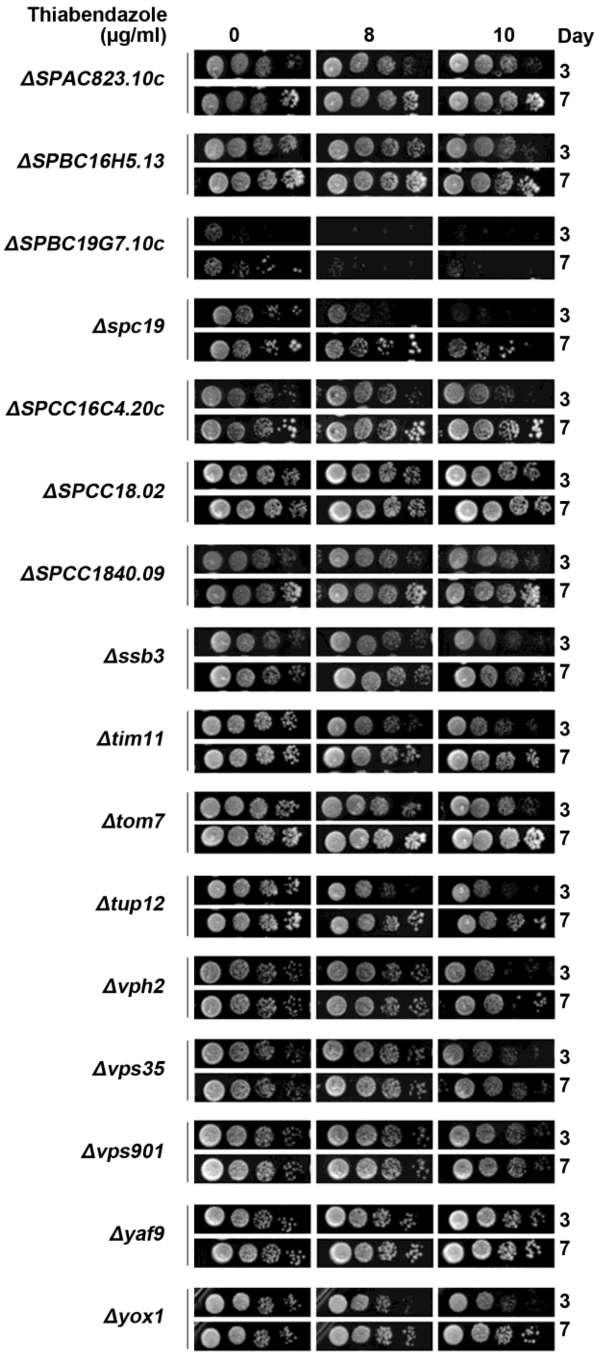
Thiabendazole
(μg/ml)
0
8
10
Day
ΔSPAC823.10c
ΔSPBC16H5.13
ΔSPBC19G7.10c
Δspc19
ΔSPCC16C4.20c
ΔSPCC18.02
ΔSPCC1840.09
Δssb3
Δtim11
Δtom7
Δtup12
Δvph2
Δvps35
Δvps901
Δyaf9
Δyox1
3
7

Supplementary Figure 5

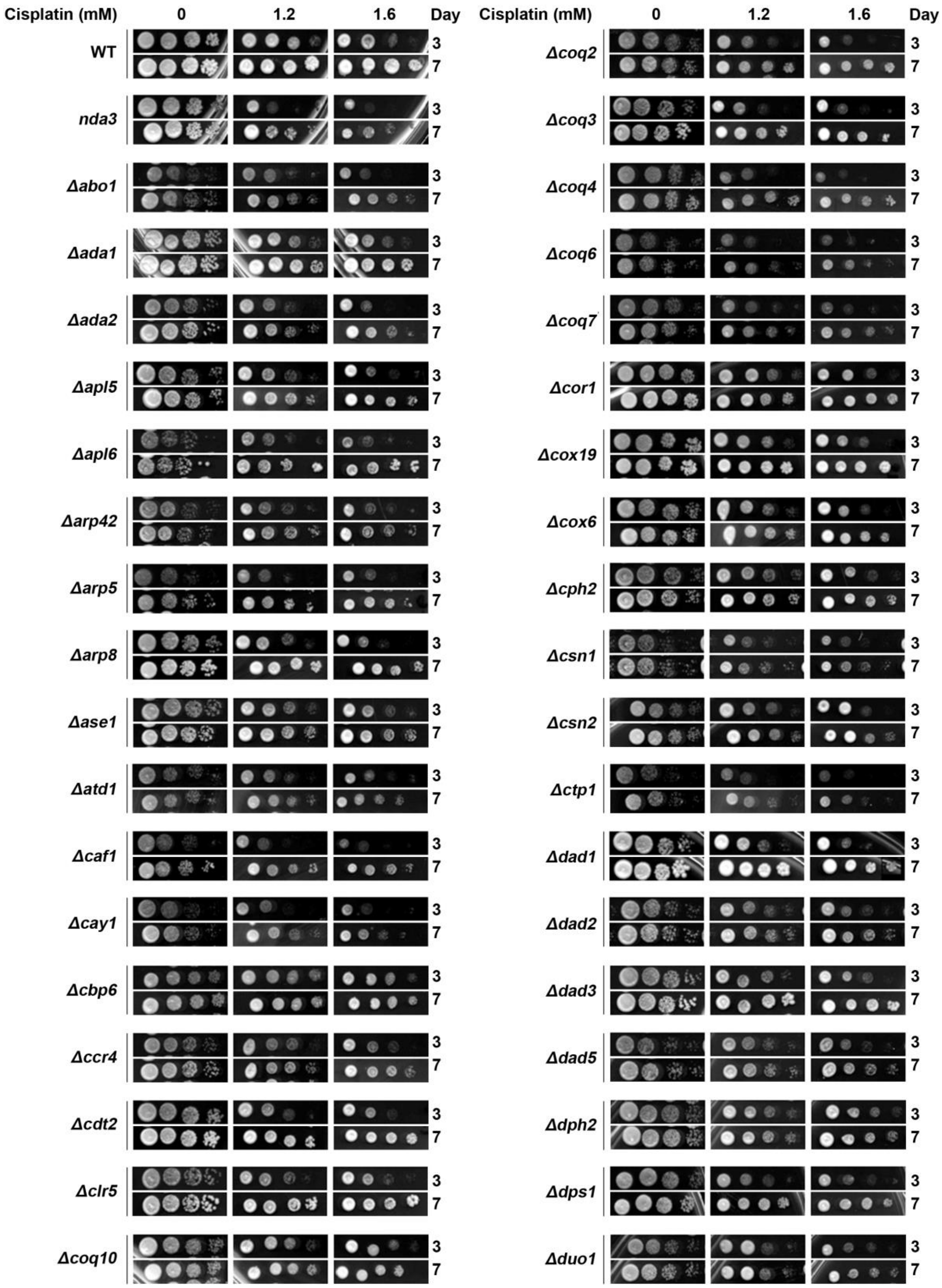

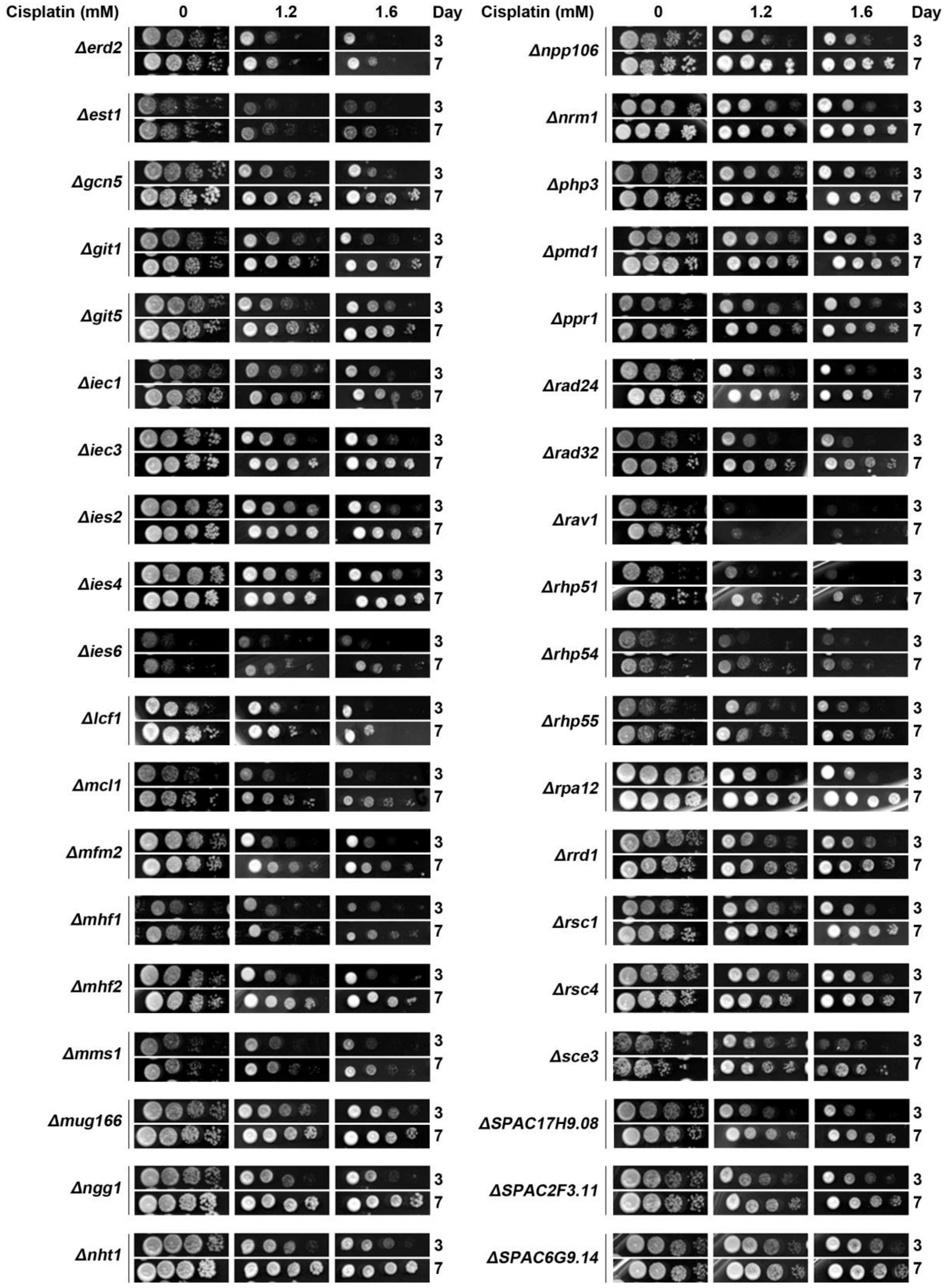

Cisplatin (mM)
0
1.2
1.6
Day
Δerd2
Δest1
Δgcn5
Δgit1
Δgit5
Δiec1
Δiec3
Δies2
Δies4
Δies6
Δlcf1
Δmcl1
Δmfm2
Δmhf1
Δmhf2
Δmms1
Δmug166
Δngg1
Δnht1
Δnpp106
Δnrm1
Δphp3
Δpmd1
Δppr1
Δrad24
Δrad32
Δrav1
Δrhp51
Δrhp54
Δrhp55
Δrpa12
Δrrd1
Δrsc1
Δrsc4
Δsce3
ΔSPAC17H9.08
ΔSPAC2F3.11
ΔSPAC6G9.14
3
7

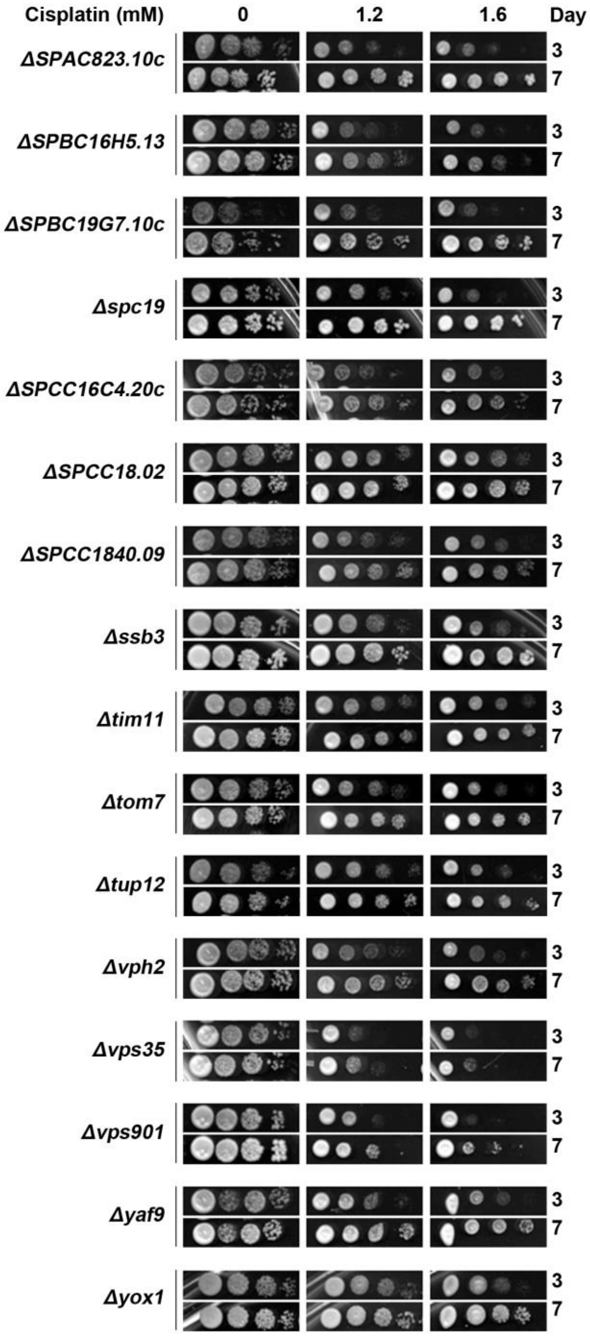
Cisplatin (mM)
0
1.2
1.6
Day
ΔSPAC823.10c
ΔSPBC16H5.13
ΔSPBC19G7.10c
Δspc19
ΔSPCC16C4.20c
ΔSPCC18.02
ΔSPCC1840.09
Δssb3
Δtim11
Δtom7
Δtup12
Δvph2
Δvps35
Δvps901
Δyaf9
Δyox1
3
7

Supplementary Figure 6

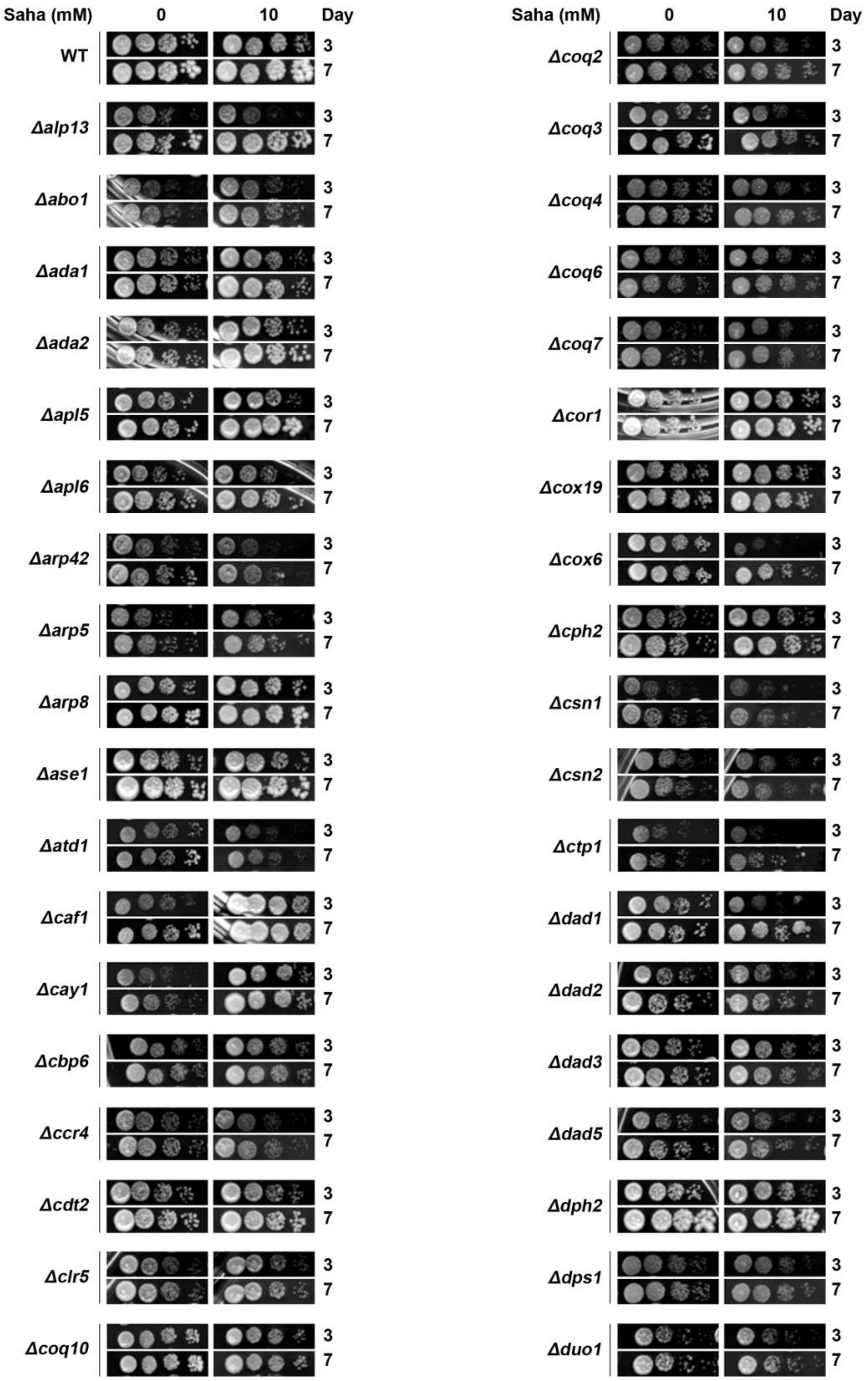

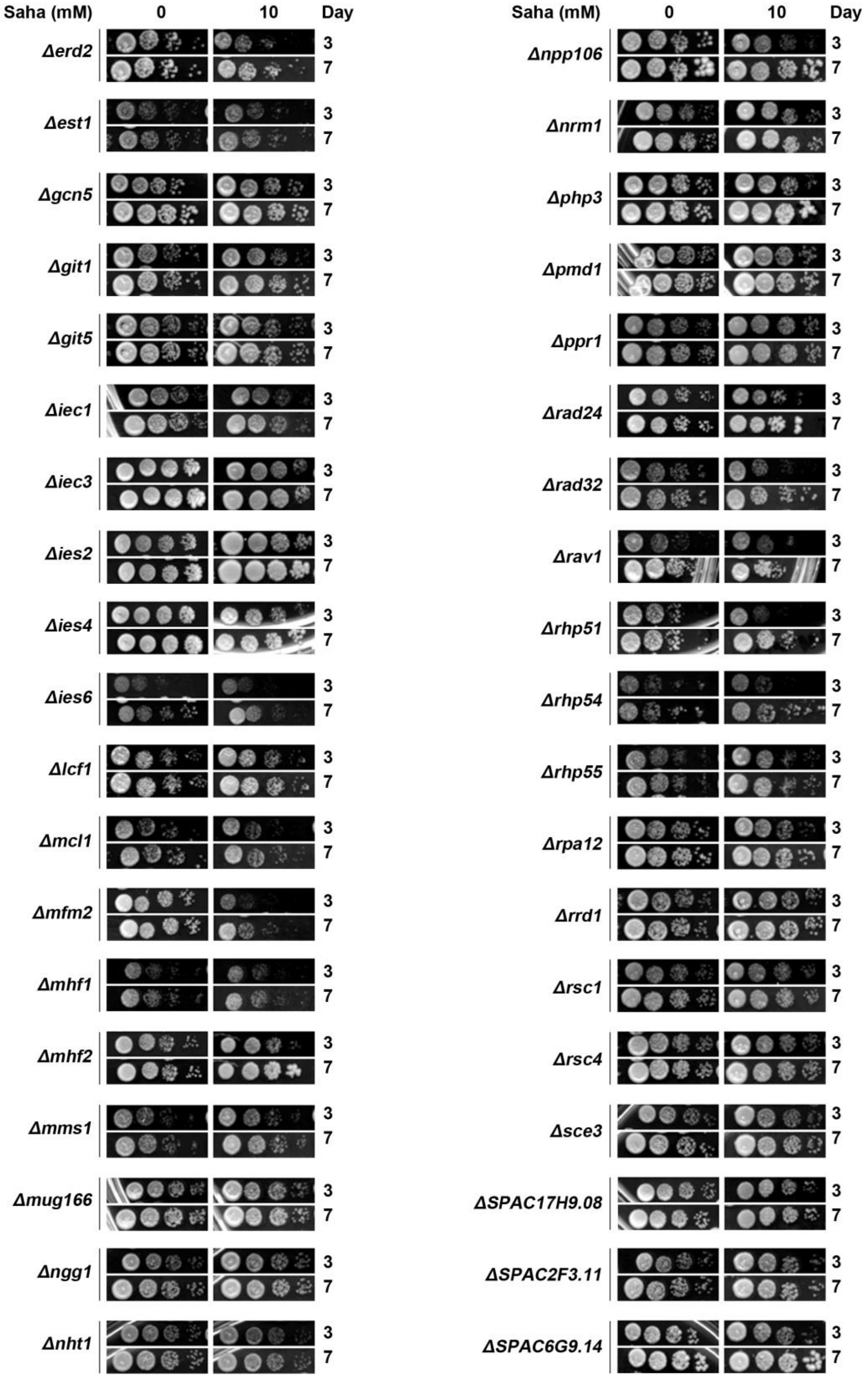
Saha (mM)
0
10
Day
3
7
Δerd2
Δest1
Δgcn5
Δgit1
Δgit5
Δiec1
Δiec3
Δies2
Δies4
Δies6
Δlcf1
Δmcl1
Δmfm2
Δmhf1
Δmhf2
Δmms1
Δmug166
Δngg1
Δnht1
Saha (mM)
0
10
Day
Δnpp106
Δnrm1
Δphp3
Δpmd1
Δppr1
Δrad24
Δrad32
Δrav1
Δrhp51
Δrhp54
Δrhp55
Δrpa12
Δrrd1
Δrsc1
Δrsc4
Δsce3
ΔSPAC17H9.08
ΔSPAC2F3.11
ΔSPAC6G9.14

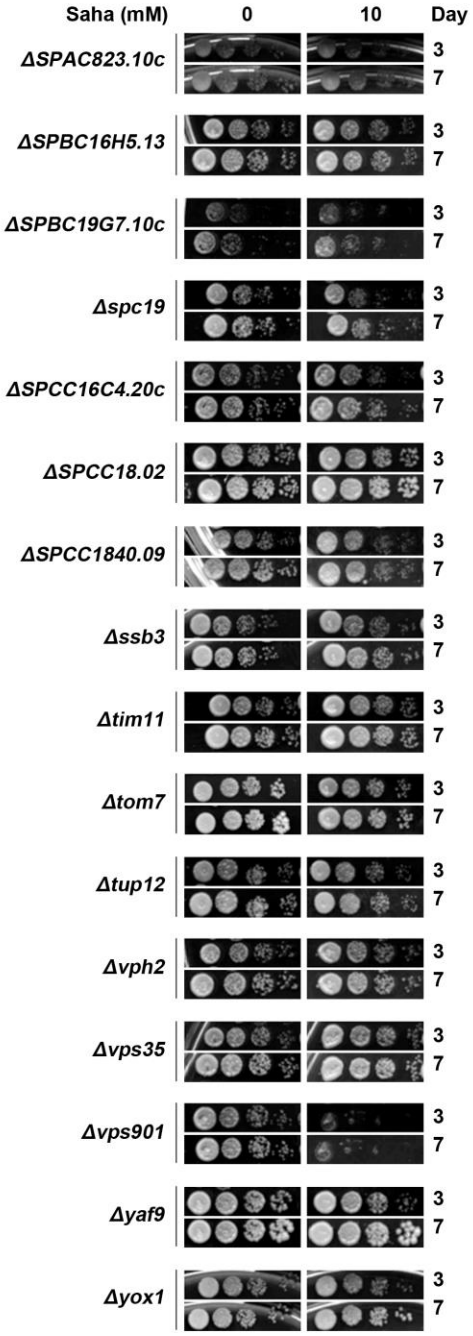

Saha (mM)
0
10
Day
ΔSPAC823.10c
ΔSPBC16H5.13
ΔSPBC19G7.10c
Δspc19
ΔSPCC16C4.20c
ΔSPCC18.02
ΔSPCC1840.09
Δssb3
Δtim11
Δtom7
Δtup12
Δvph2
Δvps35
Δvps901
Δyaf9
Δyox1
3
7

Supplementary Figure 7

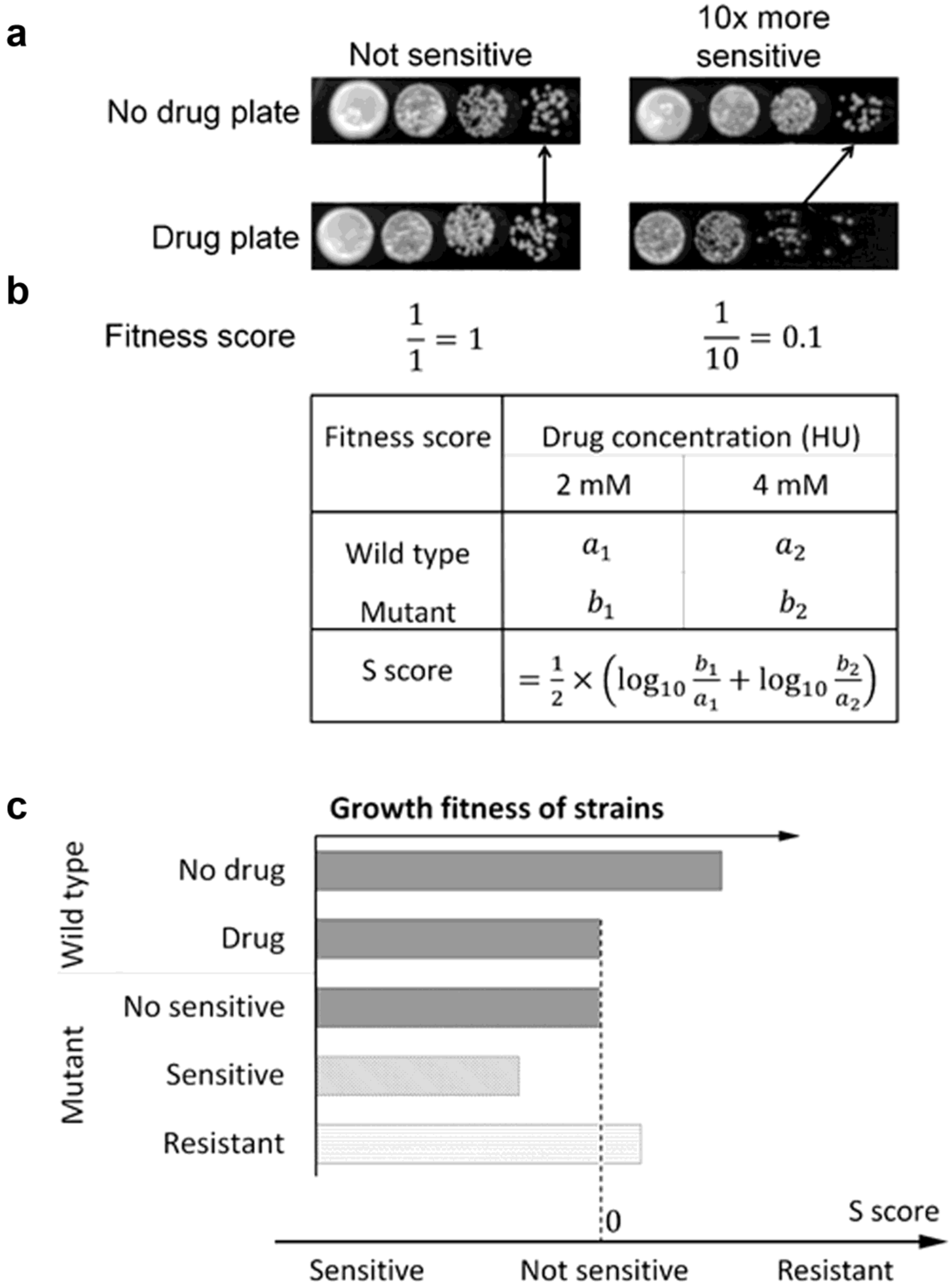

Supplementary Figure 8

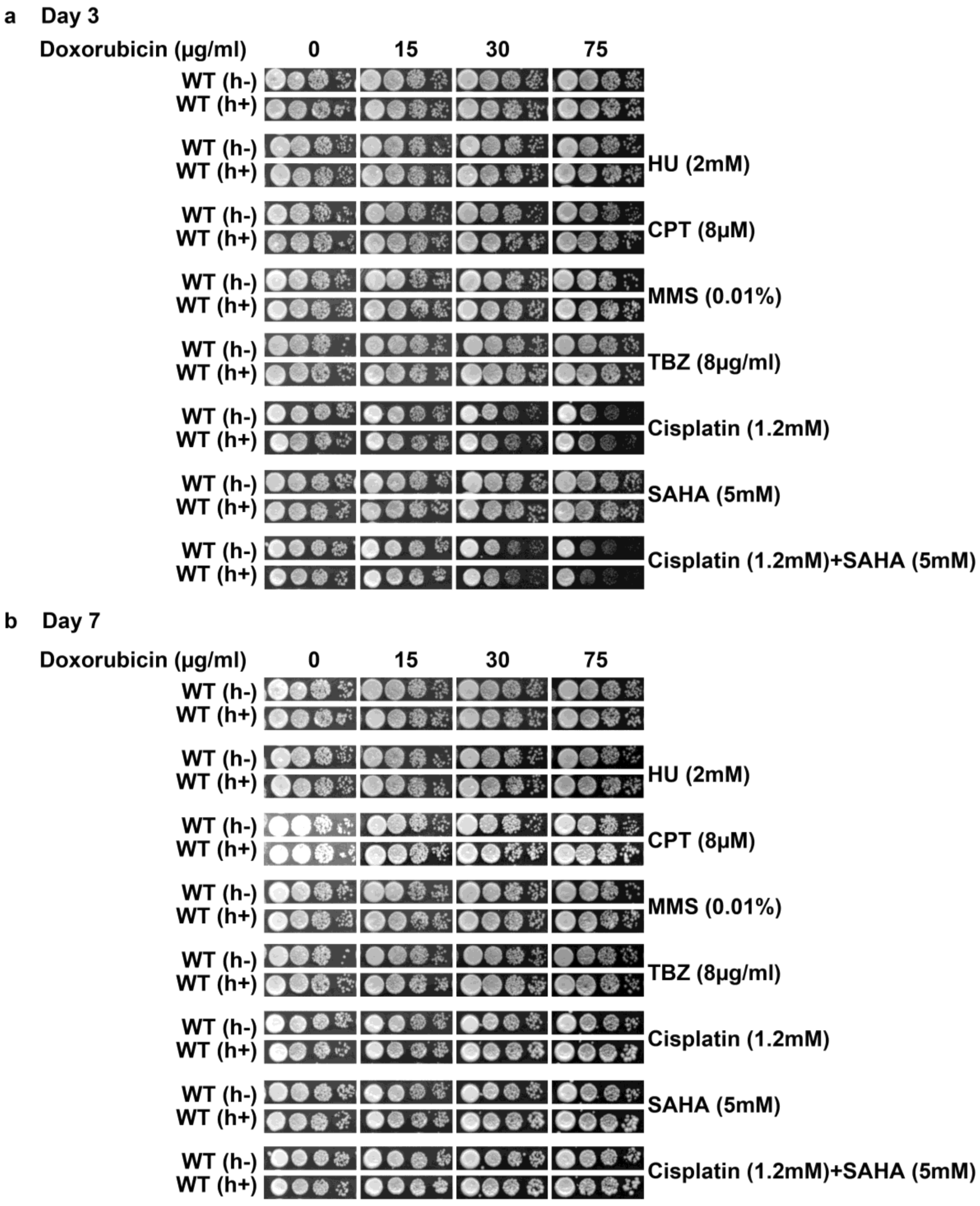

Supplementary Figure 9

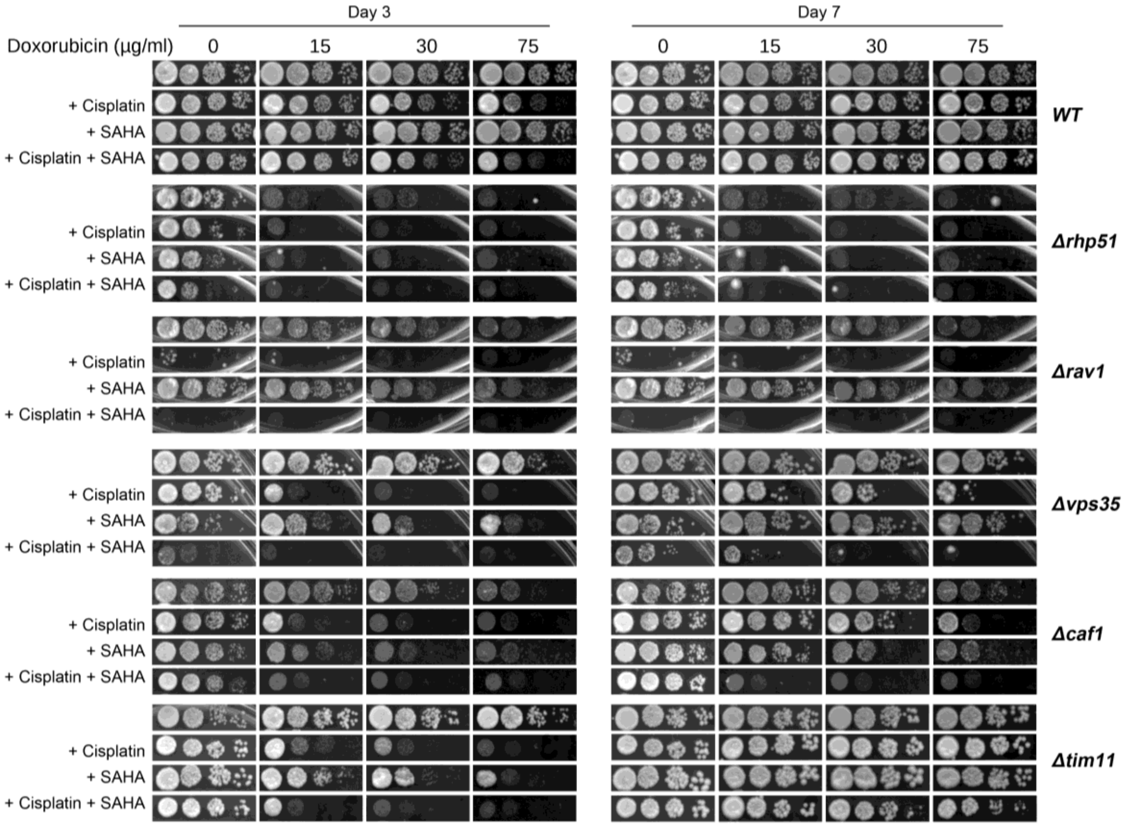

Supplementary Figure 10

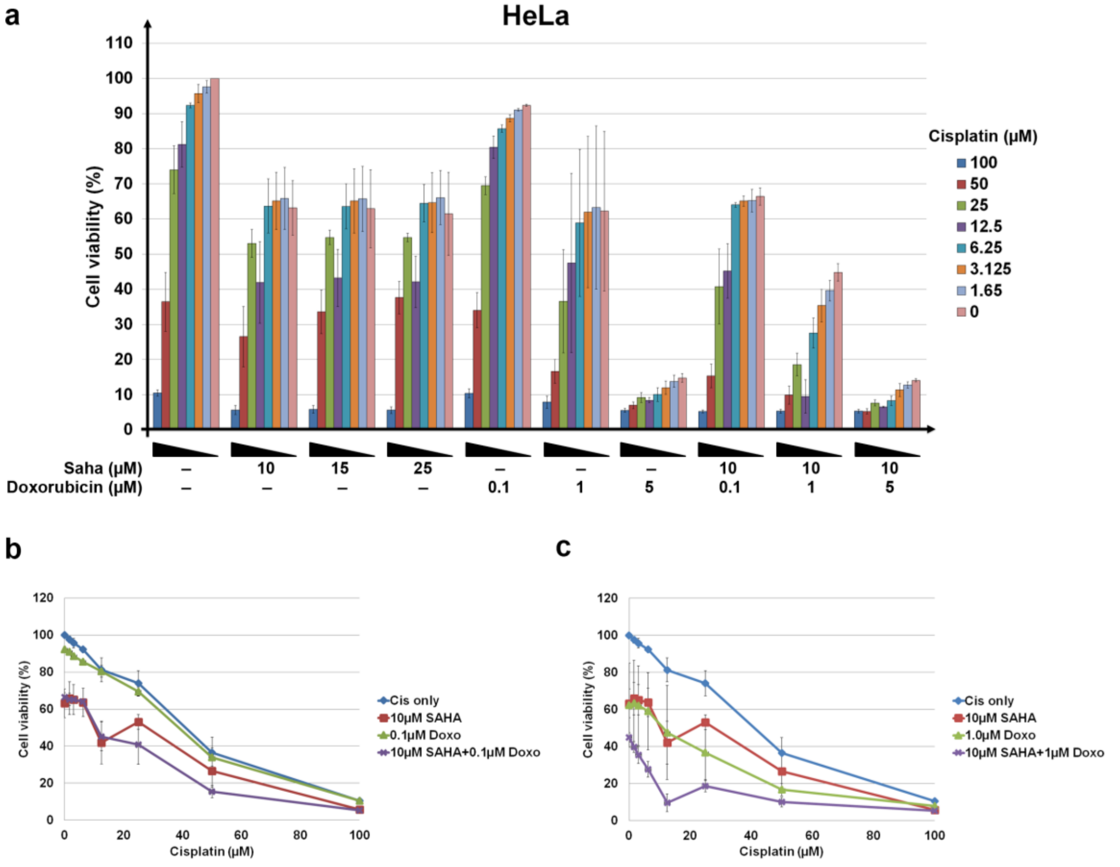

Supplementary Figure 11

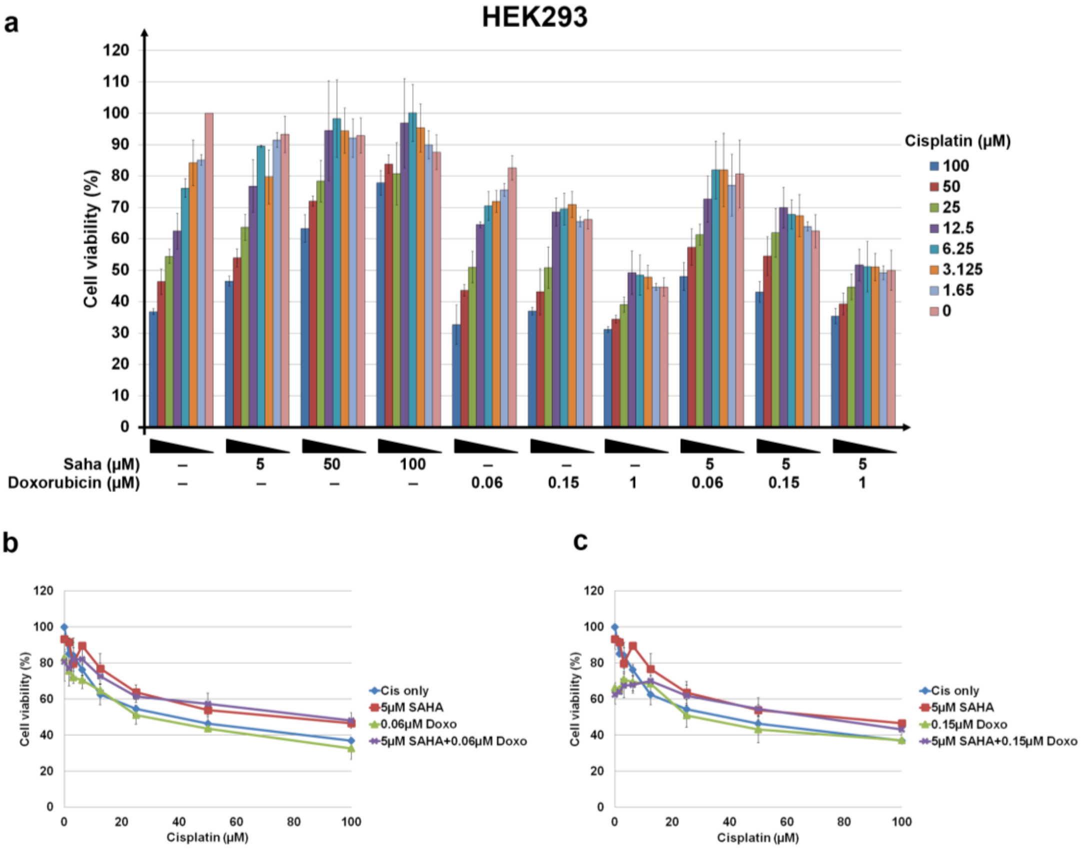

Supplementary Materials and Methods

Chemical Synthesis of SAHA

Unless stated otherwise, all non-aqueous reactions were performed in dried, round-bottomed flasks under an inert nitrogen atmosphere. Commercially available, AR-grade dichloromethane (DCM) and methanol (MeOH) were used as received. All reaction temperatures stated in the procedures are external bath temperatures. Commercial reagents were purchased from Sigma-Aldrich or Alfa Aesar, and used as received without further purification, unless otherwise stated. Yields refer to chromatographically and spectroscopically homogeneous materials, unless otherwise stated. Reaction progress was monitored by analytical thin layer chromatography (TLC) with 0.25 mm E. Merck pre-coated silica gel plates (60F-254) used UV light (254 nm) for visualization, and ceric ammonium molybdate or potassium permanganate solutions as developing stains. Flash chromatography was performed on silica gel 60 (0.040 – 0.063 mm) purchased from SiliCycle or Merck. ^{1}H NMR and ^{13}C NMR spectra were recorded on a Bruker AMX400 (400 MHz) NMR spectrometer at ambient atmosphere. The deuterated solvent used was $CDCl_3$ unless otherwise stated. Chemical shifts are reported in parts per million (ppm), and residual undeuterated solvent peaks were used as internal references: proton (7.26 ppm for $CDCl_3$, 2.50 ppm for DMSO-*d*6), carbon (77.0 ppm for $CDCl_3$, 39.52 ppm for DMSO-*d*6). ^{1}H NMR coupling constants (*J*) are reported in Hertz (Hz), and multiplicities are presented as follows: s (singlet), d (doublet), t (triplet), m (multiplet), dd (doublet of doublet), and br (broad). Low resolution mass spectra were obtained on an Agilent 6130B Quadrupole LC/MS in ESI mode.

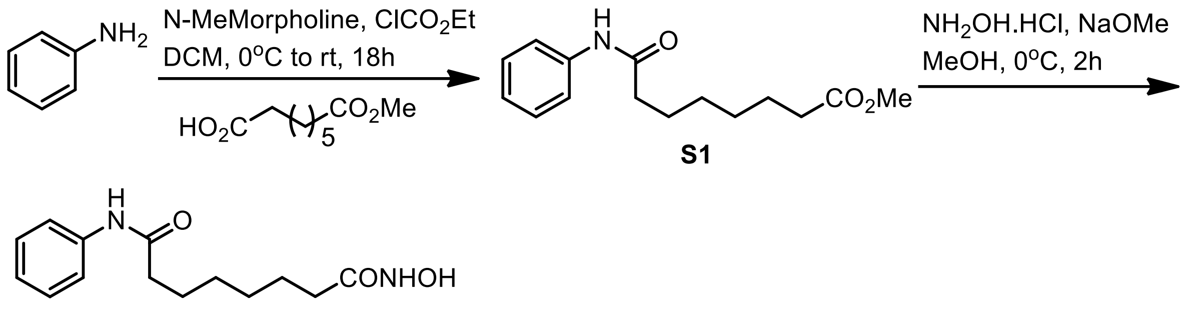

SAHA, 1

1.5 ml (8.53 mmol) of monomethyl suberate was dissolved in 30 ml of DCM and cooled to 0°C. 1.2 ml (10.9 mmol, 1.3eq.) of N-methylmorpholine and 1.0 ml (10.5 mmol, 1.2eq.) of ethyl chloroformate was added to the solution at 0°C and stirred for 15 min at 0°C. 1.0 ml (11.0mmol, 1.3eq.) of aniline was added at 0°C and the solution was allowed to warm to room temperature over 18 h. The reaction was quenched by the addition of saturated sodium bicarbonate to the solution. The mixture was extracted with DCM 3 times. The combined organic layer was washed with brine solution and dried with anhydrous sodium sulfate. The organic layer was concentrated and purified by column chromatography using 4:1 to 3:2 to 1:1 Hex/EtOAc to obtain 2.00 g (89%) of the amide as a brown solid. TLC (3:2 Hex/EtOAc) R_f = 0.31; ESI M+1 263.2

^{1}H NMR 8.23 (br. s., 1 H), 7.54 (d, J = 7.8 Hz, 2 H), 7.27 (t, J = 7.8 Hz, 2 H), 7.09 - 7.03 (m, 1 H), 3.65 (s, 3 H), 2.31 (td, J = 7.5, 17.1 Hz, 4 H), 1.74 - 1.64 (m, 2 H), 1.60 (quin, J = 7.3 Hz, 2 H), 1.37 - 1.28 (m, 4 H).

^{13}C NMR 174.2, 171.8, 138.1, 128.7, 123.9, 119.9, 51.3, 37.2, 33.8, 28.6, 28.6, 25.3, 24.5.

2.00 g (7.61 mmol) of amide **A1** was dissolved in 20 ml of methanol and cooled to 0°C. 2.68 g (38.5 mmol, 5.1eq.) of hydroxylamine hydrochloride salt and 15.0 ml (78.7 mmol, 10.3eq.) of 30% sodium methoxide solution in methanol was added to the solution. The reaction mixture was stirred for 2 h at 0°C was and then quenched with 20 ml of 4 M hydrochloric acid. The mixture was concentrated to remove methanol. The resultant precipitate was

filtered and recrystallized from methanol and water to obtain 1.66 g (82%, 74% over 2 steps) of SAHA, **1**, as an off-white crystalline solid. ESI M+1 265.2

^{1}H NMR (DMSO-d$_6$) 10.32 (br. s., 1 H), 9.83 (s, 1 H), 8.65 (br. s., 1 H), 7.58 (d, *J* = 7.5 Hz, 2 H), 7.33 - 7.23 (m, 2 H), 7.06 - 6.97 (m, 1 H), 2.28 (t, *J* = 7.5 Hz, 2 H), 1.94 (t, *J* = 7.4 Hz, 2 H), 1.64 - 1.43 (m, 4 H), 1.35 - 1.20 (m, 4 H).

^{13}C NMR (DMSO-d$_6$) δ = 171.2, 169.1, 139.3, 128.6, 122.9, 119.0, 36.4, 32.2, 28.4, 25.0.
